# Skull Ecomorphology of Megaherbivorous Dinosaurs from the Dinosaur Park Formation (Upper Campanian) of Alberta, Canada

**DOI:** 10.1371/journal.pone.0067182

**Published:** 2013-07-10

**Authors:** Jordan C. Mallon, Jason S. Anderson

**Affiliations:** 1 Department of Biological Sciences, University of Calgary, Calgary, Alberta, Canada; 2 Department of Comparative Biology & Experimental Medicine, University of Calgary, Calgary, Alberta, Canada; Ludwig-Maximilians-Universität München, Germany

## Abstract

Megaherbivorous dinosaur coexistence on the Late Cretaceous island continent of Laramidia has long puzzled researchers, owing to the mystery of how so many large herbivores (6–8 sympatric species, in many instances) could coexist on such a small (4–7 million km^2^) landmass. Various explanations have been put forth, one of which–dietary niche partitioning–forms the focus of this study. Here, we apply traditional morphometric methods to the skulls of megaherbivorous dinosaurs from the Dinosaur Park Formation (upper Campanian) of Alberta to infer the ecomorphology of these animals and to test the niche partitioning hypothesis. We find evidence for niche partitioning not only among contemporaneous ankylosaurs, ceratopsids, and hadrosaurids, but also within these clades at the family and subfamily levels. Consubfamilial ceratopsids and hadrosaurids differ insignificantly in their inferred ecomorphologies, which may explain why they rarely overlap stratigraphically: interspecific competition prevented their coexistence.

## Introduction

### Megaherbivore Diversity on Laramidia

Megaherbivorous dinosaur diversity on the Late Cretaceous island continent of Laramidia [1] was exceptionally high (particularly during the late Campanian [2]–[4]), and various metabolic, demographic, and biogeographic considerations about these animals (reviewed in Mallon et al. [5]) have caused many to wonder how so many large herbivores could coexist on such a small landmass. Two main hypotheses have traditionally been given in response to this question. One is that plant resources on Laramidia were not limiting, due to dinosaurian bradymetabolism [2], [6], [7], elevated Late Cretaceous primary productivity [7], [8], and/or predation pressure [5]. Alternatively, Laramidian plant resources may have been limiting, and megaherbivorous dinosaur coexistence was achieved via dietary niche partitioning [3], [9], [10]. This hypothesis has received little attention in the literature and is the focus of the present study.

**Figure 1:**
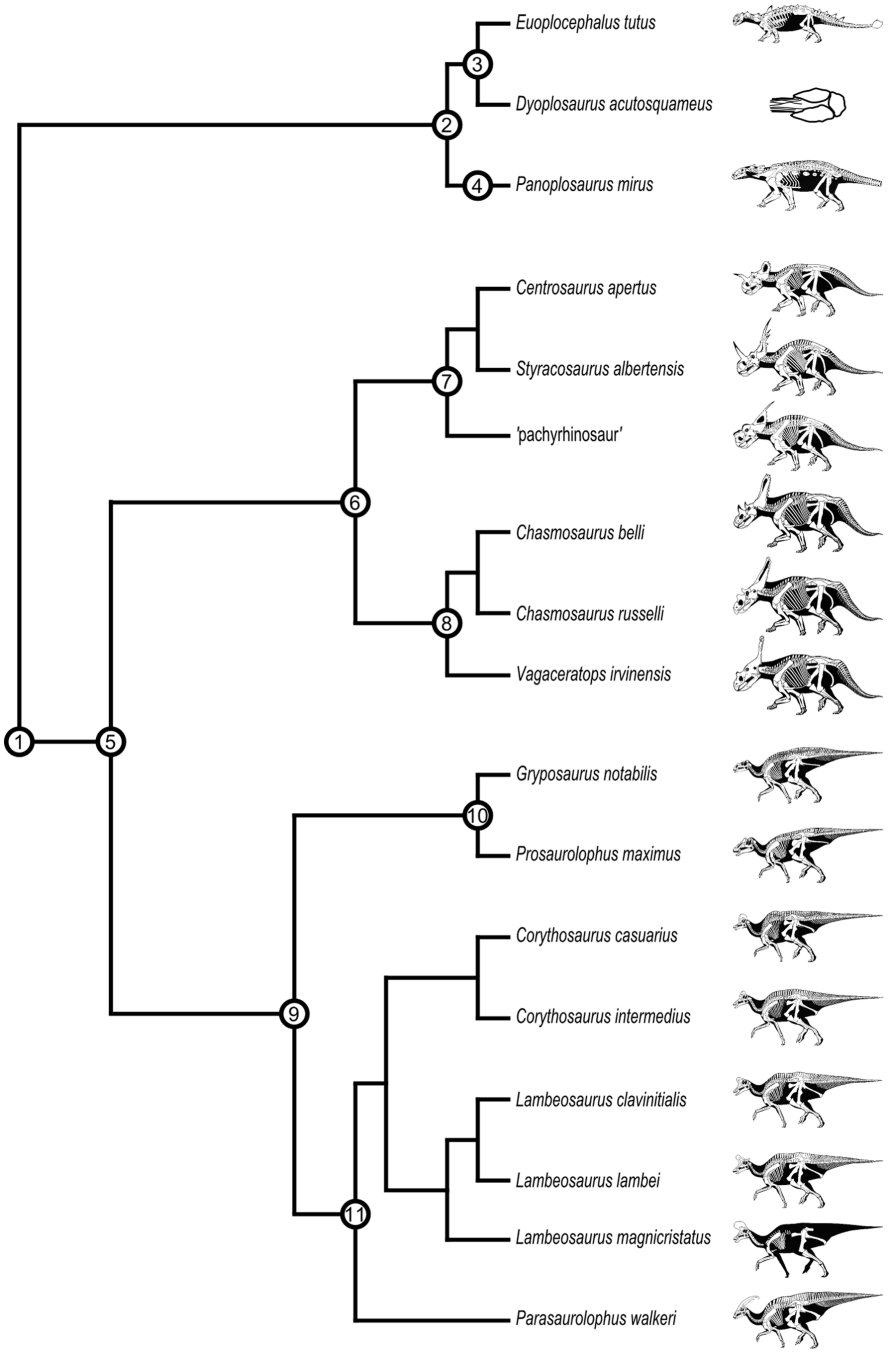
Phylogenetic relationships of megaherbivorous dinosaurs from the Dinosaur Park Formation. Suprageneric taxonomy: 1, Genasauria; 2, Ankylosauria; 3, Ankylosauridae; 4, Nodosauridae; 5, Cerapoda; 6, Ceratopsidae; 7, Centrosaurinae; 8, Chasmosaurinae; 9, Hadrosauridae; 10, Hadrosaurinae; 11, Lambeosaurinae. After Butler et al. [149], Prieto-Márquez [166], Sampson et al. [167], and Thompson et al. [168]. Skeletal drawings (not to scale) by G. S. Paul (used with permission).

The upper Campanian Dinosaur Park Formation (DPF) of Alberta is the uppermost unit of the Belly River Group, and comprises alluvial, estuarine, and paralic facies [11], [12]. We chose the megaherbivore assemblage of the DPF as a study model for three reasons: (1) the fossil record of the DPF is exceptionally rich [13]; (2) it preserves the same suite of megaherbivorous dinosaur taxa present in most time-contemporaneous strata elsewhere in western North America (e.g., ankylosaurids, nodosaurids, centrosaurines, chasmosaurines, hadrosaurines, and lambeosaurines; Figure 1); (3) its biostratigraphy is well understood [14]–[17] so that constituent taxa can be compared in biologically meaningful ways. These same considerations have influenced use of the DPF as a model in other studies of palaeoecology and taphonomy [13], [17], [18].

### Herbivore Ecomorphology

If natural selection has acted to allow vertebrate herbivores to forage optimally [19], [20] on different plants or plant parts, those herbivores should exhibit a variety of skull morphologies that correspond to variation in the plants they eat. The relationship between an organism and its environment (‘synerg’ sensu Bock and von Wahlert [21]) comprises the interaction between the biological role of some feature of that organism and the selection force acting upon it by the environment. The ecological morphology (ecomorphology) of an organism is therefore a reflection of the environment in which its parent population evolved [22]. This relationship is imperfect, owing to redundancy in the form-function complex [23] and to the confounding effects of phylogenetic inertia [24]. Nonetheless, a considerable body of work has demonstrated a fundamental relationship between herbivore skull morphology and the physical properties of the plants on which they feed (e.g., [25]–[33]).

With these principles in mind, numerous authors have suggested that dietary niche partitioning among the megaherbivorous dinosaurs from the DPF might have been facilitated via differential skull morphology [34]–[46]; however, this hypothesis has never been tested systematically. The present study seeks to test the hypothesis that the long-term coexistence of these animals was facilitated by dietary niche partitioning, with special focus given to inferring the ecomorphologies of their skulls.

### Institutional Abbreviations

All specimens were studied with permission from the following institutions: AMNH, American Museum of Natural History, New York; CMN, Canadian Museum of Nature, Ottawa; FMNH, Field Museum of Natural History, Chicago; NHMUK, Natural History Museum, London; ROM, Royal Ontario Museum, Toronto; TMM, Texas Memorial Museum, Austin; TMP, Royal Tyrrell Museum of Palaeontology, Drumheller, Alberta; UALVP, University of Alberta Laboratory of Vertebrate Palaeontology, Edmonton; USNM, National Museum of Natural History, Washington, D. C.; YPM, Yale Peabody Museum, New Haven.

## Materials and Methods

### Hypotheses

Ricklefs and Miles [47] noted that, in ecological communities shaped by the forces of competition, species overlap in morphospace tends to be minimized, reflecting the different niche requirements of the constituent species. The corollary of this is that, in communities where niche partitioning plays a negligible role, the morphological overlap of species is unconstrained.

In view of these considerations, our null hypothesis is that Late Cretaceous plant resources of Laramidia were not limiting, and that the coexistence of the megaherbivorous dinosaurs from the DPF was not facilitated by niche partitioning. In this case, we would expect to find significant overlap in morphospace, particularly between closely related species, reflecting the similar dietary niche requirements of the megaherbivores.

Our alternative hypothesis is that plant resources were limiting, and that the coexistence of the megaherbivores was facilitated by niche partitioning. If true, we would expect that species overlap in morphospace should be minimized, reflecting their different dietary niche requirements.

Ricklefs and Miles ([47]: p. 30) also emphasized that meaningful interpretations of morphospace require “a judicious selection of morphological variables reflecting a priori biomechanical function”. The morphometric model used here was therefore conceived in light of biomechanical design analyses and form-function correlations [48]–[50] observed in living herbivores. Using a combination of ordination and statistical methods (described below), we sought to quantify the degree to which megaherbivores from the DPF overlap in these morphological parameters.

### Morphometrics

**Figure 2:**
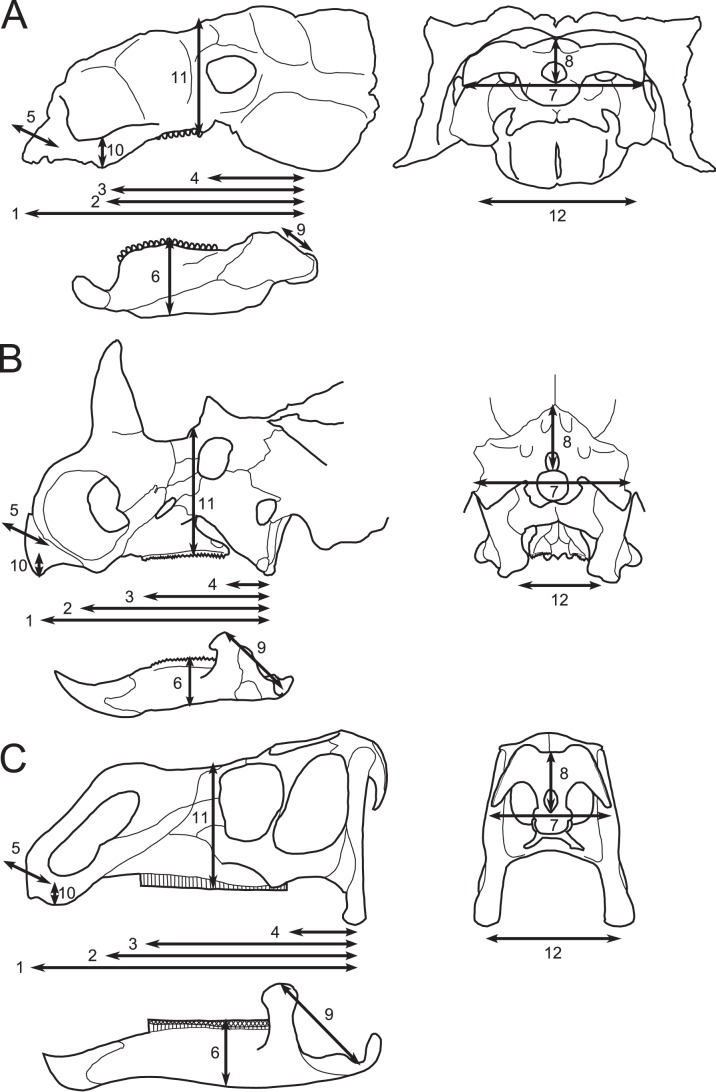
Linear measurements used in this study (compare with Table 1). A, ankylosaur skull in left lateral (left) and caudal (right) views; B, ceratopsid skull in left lateral (left) and caudal (right) views; C, hadrosaurid skull in left lateral (left) and caudal (right) views.

The ecomorphological model employed here comprises 12 linear measurements of the skull (Figure 2; Table 1), selected because of their perceived ability to reflect such aspects as plant quality, mechanical properties, and growth habit. The choice of variables stemmed from a literature pertaining to a variety of vertebrates, including lizards, turtles, birds, ungulates, macropodids, and primates. Only specimens preserving more than half of the measurements were included in the analysis to reduce the confounding effects of missing data. The total dataset (Table S1), encompassing nearly all suitable material available from the DPF, comprised 82 specimens spanning 12 megaherbivorous dinosaur genera from the clades Ankylosauria, Ceratopsidae, and Hadrosauridae, all from the DPF. The ankylosaurs *Dyoplosaurus* [51], [52] and *Scolosaurus* [53], [54], and the ceratopsid *Spinops* [55] are absent from the dataset because they lack suitable skull material (the exact provenance of *Spinops* and *Scolosaurus* are also uncertain, and may be situated in the underlying Oldman Formation). Juvenile specimens, identified by their small size and undeveloped cranial ornamentation (e.g., [16], [36]), are excluded because body size tends to be an ecologically discriminating factor [56], and their inclusion would only serve to obscure the results with respect to the question of interspecific dietary niche partitioning. Moreover, juvenile specimens are not available for all species, and selective inclusion of these specimens for some species and not others would further confound the results. We took measurements to the nearest mm with dial callipers or with a tailor’s measuring tape, where appropriate. When one side of the skull was damaged or more poorly preserved than the other, we measured only the best-preserved side; otherwise, we averaged bilateral measurements to yield a single value. In many instances, the data were multivariate non-normal, which is not ideal for use with many ordination methods [57]. We therefore log-transformed the data to produce linear relationships between variables with log-normal distributions [58], which we verified using an omnibus test for multivariate normality [59].

**Table 1:** Form-function complex of the herbivore skull (compare with Figure 1).

Variable	Functional correlate	Environmental correlate	References
1. Distance from jaw joint to rostral beak tip (SL1)	Bite force (−)	Plant mechanical resistance (−)	[44], [85], [169]–[173]
	Feeding height (−)	Plant height (−)	[27]–[29], [99]
	Feeding selectivity (+)	Plant quality (+)	[27]–[29], [99]
2. Distance from jaw joint to caudal beak tip (SL2)	Bite force (−)	Plant mechanical resistance (−)	[44], [85], [169]–[173]
	Feeding height (+)	Plant height (+)	[27]–[29], [99]
	Feeding selectivity (+)	Plant quality (+)	[27]–[29], [99], [174]
3. Distance from jaw joint to mesial endof tooth row (SL3)	Bite force (−)	Plant mechanical resistance (−)	[35], [85], [172]
4. Distance from jaw joint to distal endof tooth row (SL4)	Bite performance (−)	Plant mechanical resistance (−)	[35], [85], [172]
5. Maximum beak width (BW)	Feeding selectivity (−)	Plant quality (−)	[25], [26], [28]–[31], [99], [175]
	Feeding height (−)	Plant height (−)	[27]–[29], [99]
6. Mandible depth (MD), measured at midpointof tooth row	Accommodate cheek teeth (+)	Dietary grit (+)	[28], [31], [99]
	Adductor muscle insertion (+)	Plant mechanical resistance (+)	[27], [28], [31], [81], [83], [175]
	Resistance to bending stress (+)	Plant mechanical resistance (+)	[80], [81], [98], [99]
7. Paroccipital process breadth (PPB), measured as thesum of the lengths of the left andright paroccipital processes	Feeding height (−)	Plant height (−)	[27], [31], [99]
8. Occiput height (OH), measured from ventral edge offoramen magnum to dorsal edge of occiput	Feeding height (−)	Plant height (−)	[27], [28], [31]
9. Distance from jaw joint to coronoid processapex (JCP)	Bite force (+)	Plant mechanical resistance (+)	[35], [85], [172], [176]
10. Depression of snout below occlusal plane (SP)	Feeding height (−)	Plant height (−)	[27], [28], [31], [99]
11. Cranial height (CH), measured from baseof tooth row to dorsal surface of orbit	Resistance to bending stress (+)	Plant mechanical resistance (+)	[44]
	Bite force (+)	Plant mechanical resistance (+)	[44], [88], [96,] [172], [173], [177], [178,]
12. Distance between quadrates	Bite force (+)	Plant mechanical resistance (+)	[44], [96]

Plus (+) and minus (−) symbols indicate whether the variable and its functional and environmental correlates are positively or negatively correlated, respectively, when all other variables are held constant.

### Missing Data

As is common in palaeontology (e.g., [43]), missing data is an issue because it hinders the implementation of otherwise helpful ordination procedures used to aid the interpretation of morphometric data. Traditionally, numerous imputation methods have been applied in morphometric studies, but many of these are inadvisable [60], [61]. For example, deletion methods (e.g., listwise deletion, pairwise deletion) tend to decrease statistical power, bias parameter estimates, and lead to mathematically inconsistent matrices that are not positive definite. Similarly, many substitution methods (e.g., substitution of means, prediction by regression) lead to underestimated variances and spurious statistical significance. We therefore used the principal-component method of imputation, which accurately estimates missing values and does not suffer from the aforementioned shortcomings [60]. It works by substituting the column mean and iteratively running principal component analysis (PCA) to improve the estimates until convergence is reached. PCA allows the projection of a multivariate dataset down to a few orthogonal dimensions of maximal variance (principal components or PCs) to simplify interpretation of the data distribution [62].

### Statistical Comparisons

We drew statistical comparisons between taxonomic samples using those imputed PCA scores accounting for a significant majority (>95%) of the total variance. However, we relaxed this constraint where appropriate. We made comparisons in a hierarchical fashion at coarse (family/suborder), medium (subfamily/family), and fine (genus) taxonomic scales. We did not consider the species level because sample size was generally too low at this resolution to permit meaningful statistical comparisons. We used non-parametric multivariate analysis of variance (NPMANOVA) as a statistical test because samples were typically quite small (n <30) and non-normal. NPMANOVA tests for differences between two or more groups of multivariate data, based on any distance measure [63]. We used the Mahalanobis distance measure [64] because it is better suited to non-spherically symmetric data than the traditional Euclidean distance measure. In NPMANOVA, significance is estimated by permutation across groups, which we performed using 10,000 replicates.

We likewise conducted post-hoc pairwise comparisons using NPMANOVA with Bonferroni correction. Bonferroni correction was designed to counteract the problem of multiple comparisons, whereby the probability of committing a type I error increases with the number of simultaneous comparisons being made [58]. This problem is rectified by multiplying the *p*-value by the number of pairwise comparisons, effectively lowering the significance level. However, because Bonferroni correction provides little power and is probably too conservative [58], [65], we also report uncorrected probabilities for interpretation.

We examined those variables that best distinguish the samples using discriminant function analysis (DFA) of the imputed PCA scores. DFA is an ordination procedure whereby two or more groups of multivariate data are projected onto a reduced set of dimensions in a way that maximizes the ratio of between-group variance to within-group variance. For N groups, there are N-1 discriminant axes of diminishing importance, of which only the first few are usually informative [62]. DFA, like PCA, returns both a series of eigenvalues that indicates the amount of variation explained by each axis, and a set of loadings that denotes the importance of each variable as a discriminator along each axis. We performed all statistical and ordination procedures using the software program PAST 2.12 [57].

### Time-averaging

Because the DPF does not represent a single assemblage of contemporaneous organisms, time-averaging is an issue. This has the effect of masking palaeoecological patterns that are otherwise distinguishable only at fine temporal resolutions [66]. For this reason, we minimized the effects of time-averaging by making the above comparisons within each of the two most inclusive Megaherbivore Assemblage Zones (MAZs) identified by Mallon et al. [17]. To summarize, MAZ-1 encompasses the lower 28 m of the DPF, and MAZ-2 encompasses intervals from 29–52 m. Although this time-constrained approach theoretically increases the probability of recovering differences that would otherwise be masked by the effects of time-averaging, there is a trade-off in that sample size (and hence statistical power) is reduced considerably. Also, this approach does not completely remove the effects of time-averaging because the MAZs are themselves time-averaged over a period of approximately 600 Ka [17].

### Caveats

Dinosaurs almost certainly inhabited environments quite unlike those of the present. For example, although grasses were in existence during the Late Cretaceous [67], they did not form the extensive grasslands observed today [68]. Angiosperm-dominated forests also were quite rare. Instead, angiosperms likely took the form of herbs and shrubs growing in open and disturbed habitats [69]–[75]. As a result, conifer forests are thought to have been sparser than at present, with sunlight penetrating fully through to the ground [76]. It is therefore reasonable to ask whether modern herbivore ecomorphology should serve to inform interpretations of dinosaur palaeoecology. Two observations are offered in response to this concern. First, many modern plant genera are, in fact, known from fossil deposits of the Late Cretaceous Western Interior, including the DPF [77], [78]. It is therefore likely that many dinosaurs did consume plants similar to those alive today, despite the fact that the environments in which they lived were different in many other respects from those existing presently. Second, the independent acquisition of certain traits in response to different, but mechanically similar, plants suggests that herbivore morphology is at least partially decoupled from phylogeny and likely adheres to certain general functional principles. For example, granivores of all types [79]–[83] repeatedly evolve short skulls, deep jaws, rostrally displaced jaw adductors, and durophagous dentitions (when present); the taxonomic identity of either the granivore or the seed in question is irrelevant. Therefore, the approach taken here, whereby various aspects of palaeodiet are inferred from form-function correlations, is warranted.

## Results

The supporting ordination data (eigenvalues and variable loadings) for the results presented below are given in tables S2–S27 of Information S1.

### Time-averaged Approach

**Figure 3:**
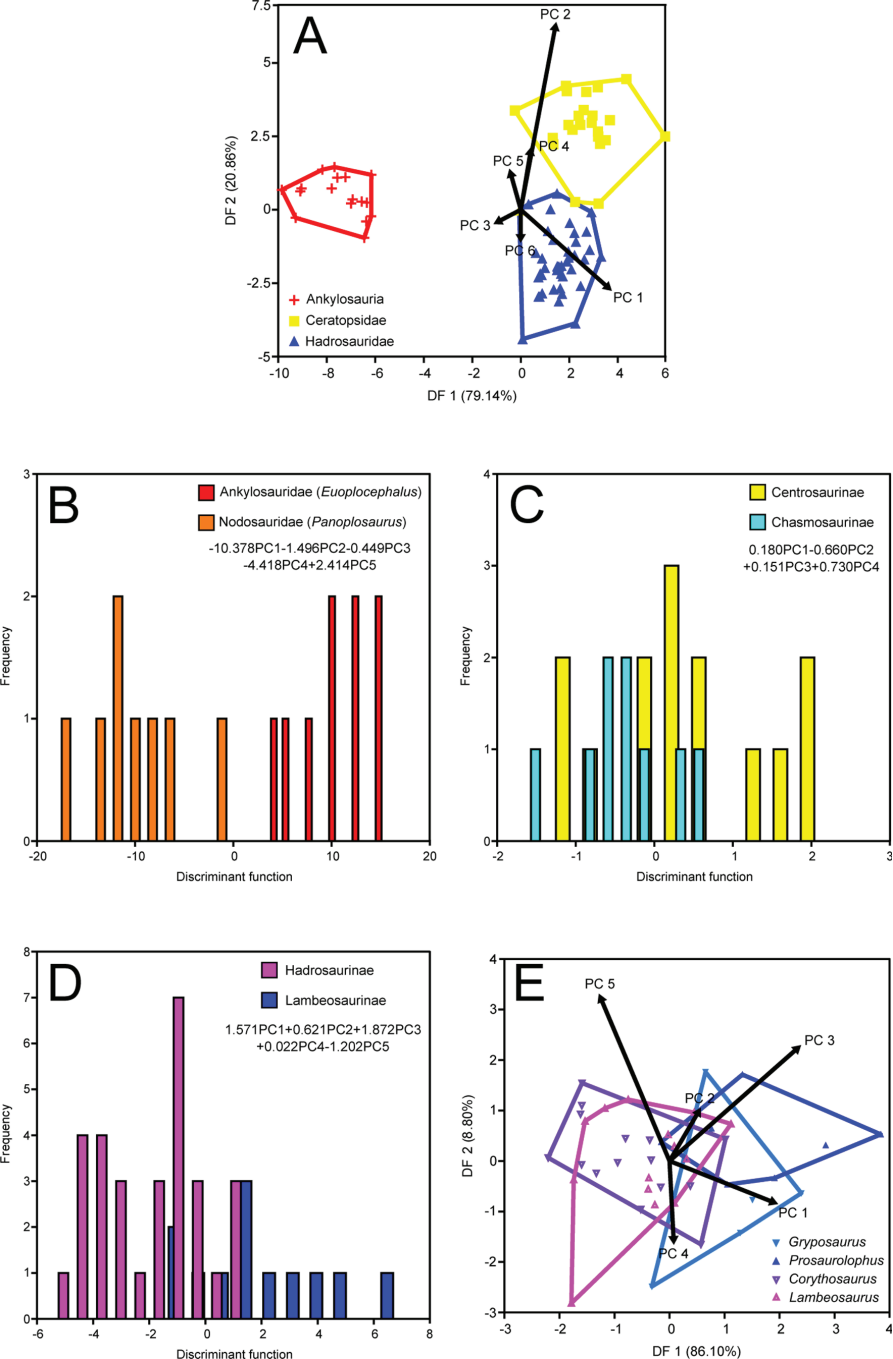
Time-averaged DFAs. A, coarse-scale analysis; B, ankylosaur family analysis; C, ceratopsid subfamily analysis; D, hadrosaurid subfamily analysis; E, hadrosaurid genus analysis.

NPMANOVA of the first six PCs reveals significant differences among the most inclusive clades (N = 82, F = 16.18, *p*<0.0001). Posthoc pairwise comparisons show that Ankylosauria, Ceratopsidae, and Hadrosauridae each differ significantly from one another (Table 2). The corresponding DFA (Figure 3A) yields a 97.56% successful classification rate. The first discriminant function (DF 1) accounts for 79.14% of the total between-group variance. Ankylosaurs score negatively on this axis, whereas ceratopsids and hadrosaurids score positively. Ceratopsids place slightly more distally on DF 1 than hadrosaurids. PC 1 loads strongly and positively on DF 1, indicating that ankylosaurs differ from ceratopsids and hadrosaurids in having smaller skulls, which are relatively broader transversely, and relatively shorter tooth rows and deeper mandibles. DF 2 accounts for the remaining between-group variance. This axis best separates ceratopsids from hadrosaurids, with ankylosaurs falling in between. PC 2 loads strongly and positively on this axis, indicating that hadrosaurids possess transversely narrower paroccipital processes and snouts with a strong ventral deflection, followed sequentially by ankylosaurs and ceratopsids.

**Table 2:** NPMANOVA results for the time-averaged coarse scale (suborder/family) taxonomic comparisons (10,000 permutations).

	Ankylosauria (n = 17)	Ceratopsidae (n = 23)	Hadrosauridae (n = 42)
Ankylosauria (n = 17)		**1.00×10^−4^**	**1.00×10^−4^**
Ceratopsidae (n = 23)	**0.0003**		**1.00×10^−4^**
Hadrosauridae (n = 42)	**0.0003**	**0.0003**	

Bonferroni corrected *p*-values shown in lower left triangle; uncorrected *p*-values shown in upper right triangle. Significant results reported in bold.

Total analysis: N = 82, F = 16.18, *p* = 1.0**×**10**^−^** ^4.^

#### Ankylosauria

The ankylosaur families Ankylosauridae and Nodosauridae (represented by *Euoplocephalus* and *Panoplosaurus*, respectively) are significantly different from one another, as revealed by NPMANOVA of the first five PCs (N = 17, F = 3.095. *p*<0.0001). The corresponding DFA perfectly discriminates *Euoplocephalus* and *Panoplosaurus* (Figure 3B). The separation is most strongly influenced by PC 1, which loads negatively on the discriminant axis. Thus, *Panoplosaurus* mainly differs from *Euoplocephalus* in having a greater offset between the jaw joint and coronoid apex.

#### Ceratopsidae

NPMANOVA of the ceratopsid subfamilies Centrosaurinae and Chasmosaurinae (chiefly represented by *Centrosaurus* and *Chasmosaurus*, respectively), using the first four PCs, produces no significant difference (N = 23, F = 1.022, *p* = 0.424). The *p*-value decreases if all 12 PCs are included in the comparison (*p* = 0.077), but otherwise remains insignificant. DFA of the first four PCs yields a 73.91% successful classification rate (Figure 3C). PC 4 loads most strongly and positively on the discriminant axis, indicating that centrosaurines generally have taller crania with slightly more distally extended tooth rows than chasmosaurines (but not significantly so). More comprehensive genus-level comparisons within subfamilies are not possible due to sample size limitations.

#### Hadrosauridae

NPMANOVA of the first five PCs yields a significant difference between the hadrosaurid subfamilies Hadrosaurinae and Lambeosaurinae (N = 42, F = 4.19, *p*<0.001). DFA yields an 88.10% successful classification rate, with lambeosaurines scoring more negatively on the discriminant axis, and hadrosaurines scoring more positively (Figure 3D). In order of decreasing magnitude, the two subfamilies are best discriminated by PCs 3 and 1, both of which load positively on the discriminant axis. Thus, hadrosaurines primarily differ from lambeosaurines in having larger skulls (PC 1) that are transversely narrower, and with less ventrally deflected beaks (PC 3).

We subjected the hadrosaurines *Gryposaurus* and *Prosaurolophus*, and the lambeosaurines *Corythosaurus* and *Lambeosaurus*, to a genus-level NPMANOVA of the first five PCs (we excluded *Parasaurolophus* due to a lack of sufficient material). We recovered significant differences among the genera (N = 41, F = 1.804, *p*<0.05). Posthoc pairwise comparisons reveal that the differences occur between lambeosaurines and hadrosaurines; there are no significant differences within these two subfamilies (Table 3). DFA of the first five PCs yields a 56.10% successful classification rate. DF 1 captures 86.10% of the total between-group variance, and DF 2 captures 8.80%. Generally, lambeosaurine genera score more negatively along DF 1, whereas hadrosaurine genera score more positively (Figure 3E). There is poor separation along DF 2. Examination of the loadings reveals that PCs 1 and 3 both load strongly and positively on DF 1, which unsurprisingly mirror the shape changes captured by the hadrosaurine-lambeosaurine analysis above.

**Table 3:** NPMANOVA results for the time-averaged hadrosaurid genus comparisons (10,000 permutations).

	*Gryposaurus* (n = 5)	*Prosaurolophus* (n = 7)	*Corythosaurus* (n = 15)	*Lambeosaurus* (n = 15)
*Gryposaurus* (n = 5)		0.7053	0.08309	**0.05389**
*Prosaurolophus* (n = 7)	1		**0.0049**	**0.0286**
*Corythosaurus* (n = 15)	0.4986	**0.0294**		0.8046
*Lambeosaurus* (n = 15)	0.3234	0.1716	1	

Bonferroni corrected *p*-values shown in lower left triangle; uncorrected *p*-values shown in upper right triangle. Significant results reported in bold.

Total analysis: N = 41, F = 1.804, *p* = 0.0245.

### MAZ-1

**Figure 4:**
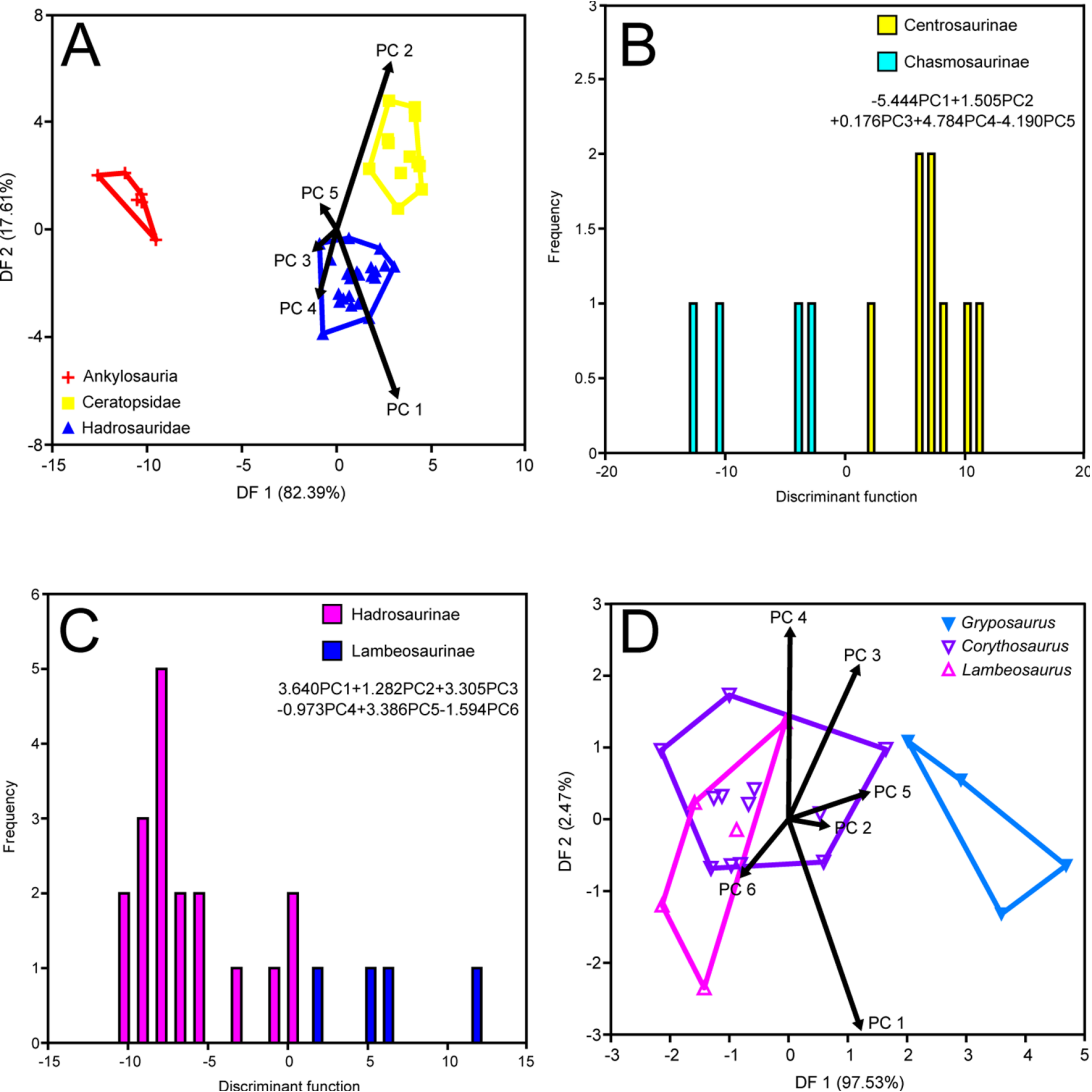
Time-constrained MAZ-1 DFAs. A, coarse-scale analysis; B, ceratopsid subfamily analysis; C, hadrosaurid subfamily analysis; D, hadrosaurid genus analysis.

NPMANOVA of the first five PCs recovers significant differences among ankylosaurs, ceratopsids, and hadrosaurids (N = 40, F = 10.33, *p*<0.0001). Posthoc pairwise comparisons demonstrate that each of these clades differs significantly from the other (Table 4). The corresponding DFA yields a 100% successful classification rate. The ordination results (Figure 4A) correspond to those of the time-averaged analysis, such that all three clades occupy similar areas of morphospace; however, there is better separation of all taxa–particularly ceratopsids and hadrosaurids–probably a reflection of the overall smaller sample size. Although the discriminant axes capture a similar amount of between-group variation as the time-averaged DFA, their loadings differ slightly. In the MAZ-1 analysis, PCs 1 and 2 load subequally on the first axis. This appears to be a consequence of the increased group separation along DF 1. PC 1 captures a similar signal to that reported for the time-averaged analysis, separating ankylosaurs from ceratopsids and hadrosaurids on the basis of skull length and breadth, tooth row length, and mandible depth. Conversely, PC 2 appears to reflect the fact that ceratopsids possess broader paroccipital processes and less ventrally deflected snouts than hadrosaurids. Evidently, some of the signal captured by DF 2 in the original, time-averaged DFA has ‘leaked’ over onto DF 1 in this analysis. PC 1 loads strongly and negatively on DF 2, whereas PC 2 loads strongly and positively. The morphological signal captured by DF 2 is similar to that captured by DF 1; the different loadings between the first two DF axes simply reflect the different relative positions of the taxa along those axes.

**Table 4:** NPMANOVA results for the MAZ-1 coarse scale (suborder/family) taxonomic comparisons (10,000 permutations).

	Ankylosauria (n = 6)	Ceratopsidae (n = 12)	Hadrosauridae (n = 22)
Ankylosauria (n = 6)		**1.00×10^−4^**	**1.00×10^−4^**
Ceratopsidae (n = 12)	**0.0003**		**1.00×10^−4^**
Hadrosauridae (n = 22)	**0.0003**	**0.0003**	

Bonferroni corrected *p*-values shown in lower left triangle; uncorrected *p*-values shown in upper right triangle. Significant results reported in bold.

Total analysis: N = 40, F = 10.33, *p* = 1.0**×**10**^−^** ^4.^

#### Ankylosauria

We did not conduct statistical comparisons of ankylosaurs due to sample size limitations.

#### Ceratopsidae

Centrosaurines and chasmosaurines (represented solely by *Centrosaurus* and *Chasmosaurus*, respectively) cannot be distinguished from one another using NPMANOVA of the first four PCs (N = 12, F = 1.799, *p*>0.05), but the two taxa are significantly different with the inclusion of PC 5 (N = 12, F = 1.92, *p*<0.05). DFA using the first five PCs yields a 100% successful classification rate, but given the particularly small chasmosaurine sample (n = 4), this result may be artificially inflated. Chasmosaurines score negatively on the discriminant axis, and centrosaurines score positively (Figure 4B). PC 1 loads most strongly and negatively on the discriminant axis, indicating that chasmosaurines generally possess a transversely wider occipital region of the skull. PC 4 also loads strongly and positively on the axis, reflecting the fact that centrosaurines possess a relatively wider beak and dorsoventrally deeper skulls.

#### Hadrosauridae

Hadrosaurines and lambeosaurines are significantly different (N = 22, F = 2.586, *p*<0.01), as revealed by NPMANOVA of the first six PCs. DFA results in a 95.45% successful classification rate. Lambeosaurines generally score negatively on the discriminant axis, whereas hadrosaurines score positively (Figure 4C). PC 1 loads most strongly and positively on the discriminant axis, indicating that hadrosaurines possess larger skulls with slightly wider occipital regions than lambeosaurines. PCs 3 and 5 also load strongly and positively on the discriminant axis, but their signals are more difficult to interpret because their loadings sometimes conflict. Both PCs reveal that hadrosaurines have relatively narrower snouts than lambeosaurines.

We included *Gryposaurus*, *Corythosaurus*, and *Lambeosaurus* in a genus level comparison. No significant differences are recovered among the genera (N = 21, F = 1.437, *p*>0.05), but the posthoc pairwise comparisons do support the contention that hadrosaurines and lambeosaurines tend to be most different (Table 5). DFA yields a 71.43% successful classification rate. The ordination and loadings correspond to those of the subfamily comparisons (Figure 4D).

**Table 5:** NPMANOVA results for the MAZ-1 hadrosaurid genus comparisons (10,000 permutations).

	*Gryposaurus* (n = 4)	*Corythosaurus* (n = 12)	*Lambeosaurus* (n = 6)
*Gryposaurus* (n = 4)		**0.0171**	0.195
*Corythosaurus* (n = 12)	**0.05129**		0.9403
*Lambeosaurus* (n = 6)	0.5849	1	

Bonferroni corrected *p*-values shown in lower left triangle; uncorrected *p*-values shown in upper right triangle. Significant results reported in bold.

Total analysis: N = 21, F = 1.437, *p* = 0.1211.

### MAZ-2

**Figure 5:**
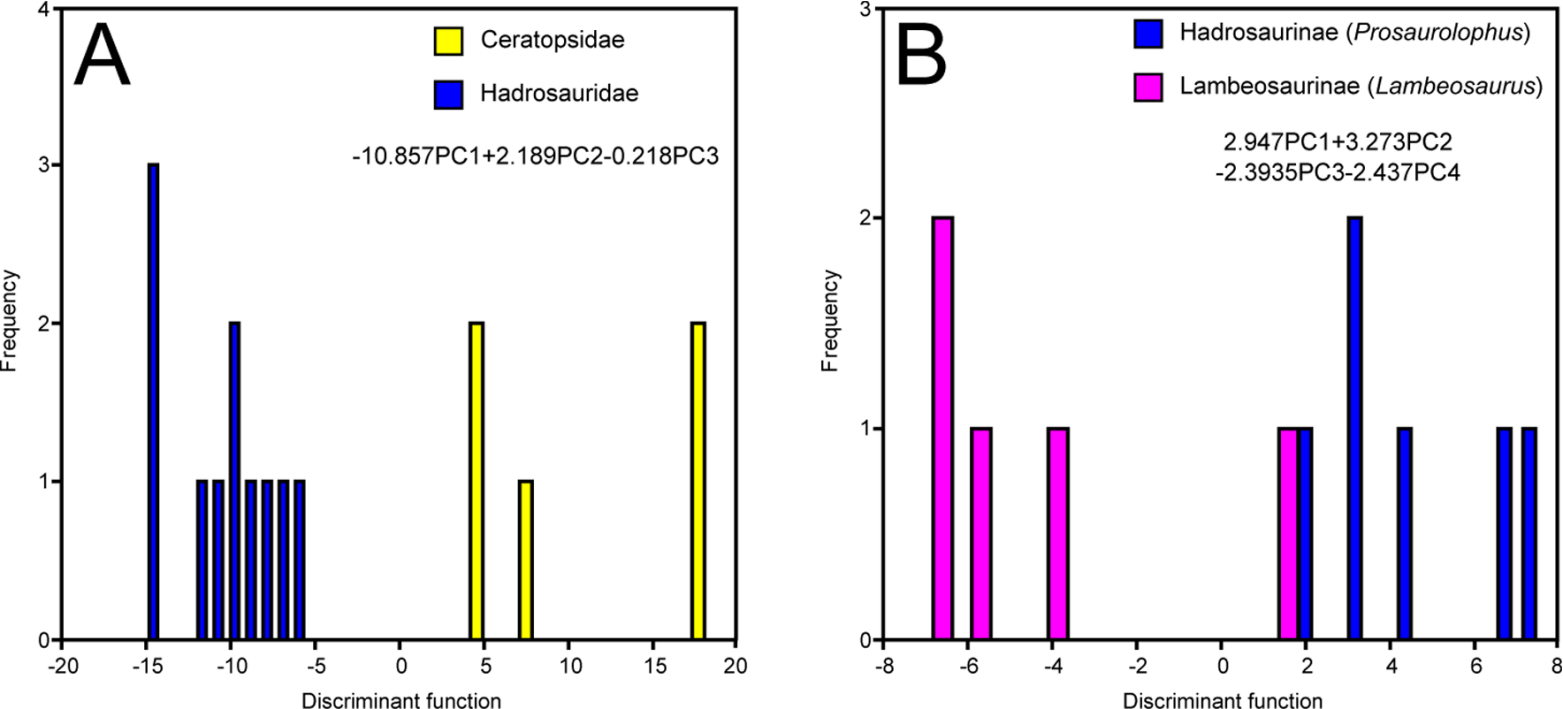
Time-constrained MAZ-2 DFAs. A, coarse-scale analysis; B, hadrosaurid analysis.

The MAZ-2 analyses do not include ankylosaurs due to sample size limitations in this interval [17]. NPMANOVA of the first three PCs yields a highly significant difference between ceratopsids and hadrosaurids (N = 16, F = 5.434, *p* = 0.001). Both taxa are perfectly discriminated using DFA. Hadrosaurids score negatively on the discriminant axis, whereas ceratopsids score positively (Figure 5A). This separation is most influenced by PC 1, which correlates negatively with the discriminant axis. Thus, hadrosaurids are distinguished from ceratopsids primarily by their ventrally deflected rostra and transversely narrow skulls.

#### Ceratopsidae

We did not conduct statistical comparisons of ceratopsids due to sample size limitations.

#### Hadrosauridae

Hadrosaurines and lambeosaurines (represented by *Prosaurolophus* and *Lambeosaurus*, respectively) are significantly different, as revealed by NPMANOVA of the first four PCs (N = 11, F = 2.026, *p*<0.05). DFA results in a 90.91% successful classification rate. Lambeosaurines score more negatively on the discriminant axis, and hadrosaurines score more positively (Figure 5B). PC 1 loads most strongly and positively on this axis, reflecting the larger skull size of *Prosaurolophus* relative to *Lambeosaurus*.

## Discussion

### Palaeodietary Implications

#### Ankylosauria

The inferred ankylosaur ecomorph is characterized by a small, proportionally wide skull, with a relatively deep mandible and short tooth row. The snout is ventrally deflected, but not as strongly as in hadrosaurids. First-hand examination of ankylosaur specimens reveals that the depth of the mandible is exaggerated by the dorsal bowing of the tooth row (Figure 2A). Ankylosaurs are not strongly distinguished from either ceratopsids or hadrosaurids based on the distance between the jaw joint and coronoid process apex. This is somewhat surprising because both ceratopsids and hadrosaurids possess elevated coronoid processes and depressed jaw joints that are otherwise not developed to the same degree in ankylosaurs [84]–[86]. It may be that the ankylosaur coronoid apex is more rostrally displaced than in ceratopsids and hadrosaurids, resulting in subequal measurements of this variable. Further work illuminating the differences in jaw mechanics between these taxa is in progress.

Because jaw adductor muscle mass–and by extension, bite force–generally scales positively with skull size (e.g., [87]–[91]), it is likely that ankylosaurs possessed a weaker bite than the larger ceratopsids and hadrosaurids. Likewise, the rostral placement of the tooth row relative to the coronoid process in ankylosaurs means that they did not possess as powerful a bite as the other two taxa, in which the tooth row extends caudal to the coronoid process, resulting in increased leverage of the distal tooth row [84]–[86]. Other evidence cited in favour of a relatively weak bite in ankylosaurs is the presence of small, phyliform teeth with peg-like roots [92]–[94], and simple jaw musculature [95].

Nonetheless, the ankylosaur skull exhibits other features thought to correlate with either high bite forces or repetitive masticatory movements–both adaptations for comminuting resistant plant matter. For example, the proportionally great transverse breadth of the skull may have accommodated larger jaw adductor muscles. This explanation was offered by Herrel et al. [96] to account for the fact that finches with relatively wide skulls also possess the highest bite forces. Henderson [44] likewise used beam theory to show that wider skulls are able to resist high torsional stresses incurred by elevated bite forces. Furthermore, the curved tooth row of ankylosaurs is reminiscent of that of grazing macropodoids, the function of which Sanson [97] surmised was to concentrate bite forces in response to a tough diet. The secondary increase in the depth of the mandible likewise would have served to withstand repeated bending forces associated with mastication, preventing bone fatigue [98], [99]. Finally, Vickaryous et al. [100] cited the presence of an ossified secondary palate in ankylosaurs as evidence that their skulls were adapted to resisting strain resulting from complex jaw movements used in the comminution of tough plants.

Besides reconstructed feeding envelopes [5], several other morphological characters attest to the low-browsing habit of ankylosaurs. First is the broad, ventrally-deflected snout, which is otherwise observed most frequently among grazing bovids [99]. The relatively great breadth of the snout undoubtedly enables these mammals to feed more efficiently on fibrous, low-growing grasses [25]–[32]. However, the purpose of the ventral deflection of the snout in these bovids is not yet fully understood. It may serve to bring the cropping mechanism (incisors) closer to the ground, similar to what has been proposed for marine grazing dugongs [101], but this is speculation. It may also reflect the fact that grazers tend to have faces more strongly flexed on the basicranium than browsers [27], [28], [31], but the reason for this correlation is likewise unknown.

Second is the relatively great transverse breadth of the paroccipital processes, which is also common among grazing bovids. Spencer [99] suggested that this may reflect the fact that grazers tend to use sharp head movements for cropping forage, effected by the nuchal musculature, whereas browsers rely more on their lips and tongue. Perhaps ankylosaurs also relied on head movements to sever plant food, but no corresponding study on head mobility in these animals has been conducted to date. Challenging this hypothesis is the observation of Maryańska [102] that ankylosaurs possessed a well-developed hyoid apparatus and entoglossal process, which would have supported a long and mobile tongue. Ankylosaurs may have used such a tongue in the cropping of vegetation.

Therefore, while it is likely that they consumed soft, pulpy plant tissues (e.g., fruits [93], [103]), ankylosaurs probably subsisted on tough leaves that required more thorough mastication as well [104]. This interpretation is corroborated by circumstantial evidence in the form of a cololite associated with a Lower Cretaceous ankylosaurid from Australia [105]. The fossil comprises angiosperm fruits or endocarps, small seeds, possible fern sporangia, and abundant vascular tissue (probably leaves). The plant material exhibits signs of having been comminuted by the jaws [105]; however, the finding of gastroliths associated with a specimen of *Panoplosaurus mirus* (ROM 1215 [106]) suggests that additional food processing occurred in the gizzard. If so, then it is likely that the ankylosaur skull ecomorph does not accurately reflect the associated palaeodiet. Nonetheless, there is some doubt about whether the gastroliths truly pertain to the specimen in question, as neither the field notes nor the original description [92] mention the existence of gizzard stones (K. Seymour, pers. comm., 2011).

Ankylosaur families differ in their mandibular morphologies such that nodosaurids possess a relatively greater offset between the jaw joint and coronoid apex than ankylosaurids. This suggests that the mechanical advantage of the nodosaurid mandible was elevated relative to that of ankylosaurids (due to the increased length of the applied force moment arm), resulting in a more powerful bite. Supporting this interpretation, Carpenter [106] and Vickaryous [107] reported on the existence of dorsoventrally deep (fused) vomers with a distally dilated process among nodosaurids, which may have served to further dissipate stress associated with either elevated bite forces or repetitive masticatory movements. Therefore, it seems likely that nodosaurids subsisted on harder or tougher plants than ankylosaurids, necessitating a more powerful bite and cranial structures associated with stress distribution.

Perhaps surprisingly, the contention of Carpenter [38]–[41] that ankylosaurids and nodosaurids differ appreciably in relative beak width is not well-supported here. In the time-averaged ankylosaur analysis above, the separation of the two families along PC 1 is due in part to the relatively wider beak of ankylosaurids, but this variable loads comparatively weakly on the axis, and its signal is otherwise contradicted by loadings on other PCs. It is possible that relative beak width did not prove to be a stronger discriminator of ankylosaurids and nodosaurids because: (1) it was overwhelmed by other, stronger loading variables; (2) it was not captured by the first PCs considered here; or (3) it was not captured at all due to the confounding effects of missing data. Additional research into the specific question of ankylosaur beak width variation is in progress.

#### Ceratopsidae

The inferred ceratopsid ecomorph is characterized by a particularly large and narrow skull, distally-elongate tooth row, and rostrally projecting snout. Although the relative transverse width of the paroccipital processes is most developed in ankylosaurs, the separation of ceratopsids from hadrosaurids along DF 2 of the time-averaged analysis suggests that the former taxon is characterized by slightly wider paroccipital processes as well.

Two features in particular attest to the especially powerful bite of ceratopsids. The first is overall skull size, which is the largest of any of the forms from the DPF. The second is the distal extension of the tooth row beyond the apex of the coronoid process. Ostrom [35], [85] demonstrated that this morphology equates to a shift in the behaviour of the jaw mechanism, from a class 3 to a class 1 lever, because the relative lengths of the applied and resistance force moment arms are switched. Therefore, the ceratopsid jaw mechanism appears to have been more efficient than that of ankylosaurs. The elevation of the coronoid process and concomitant depression of the jaw joint would have further served to enhance the leverage of the ceratopsid mandible [35], [85].

The transversely wide paroccipital processes of ceratopsids–although not as developed as in ankylosaurs–may correlate with low browsing. On the other hand, it may reflect the development of the nuchal musculature in support of the large parietosquamosal frill. Paradoxically, although ceratopsids appear to have been restricted to feeding below one metre from the ground [5], the cropping mechanism is not ventrally deflected as in mammalian grazers [99]. This might be attributable to the great mobility of the head, which could have pivoted easily about the spherical occipital condyle to bring the beak near to the ground [108].

Bearing these points in mind, ceratopsids can be characterized as low-level browsers that probably sustained themselves on mechanically resistant vegetation requiring high bite forces. Mechanical resistance comprises various physical properties such as strength, toughness, and ‘hardness’ (a general term that encompasses the properties of plasticity and stiffness). The bladed dentition of ceratopsids [34], [35], [85], [109]–[111] almost certainly was not suitable for processing particularly strong or hard plant types, which require a durophagous dentition [82]. Therefore, it is likely that ceratopsids specialized on tough plant parts that resisted crack propagation, such as low-growing, woody browse. The ‘weedy’ angiosperms of the Late Cretaceous, which grew most commonly in coastal plain settings [112] alongside ceratopsids [113], may have provided an abundant and renewable food resource for these animals [114]–[116]. The interpretation of ceratopsids as woody browse specialists might help to explain the narrowness of their beaks, which would have restricted them to selective foraging, but more work in this area is required.

NPMANOVA indicates that centrosaurines and chasmosaurines probably differ in their skull proportions, but low sample size generally impedes the interpretation of the results. For example, whereas the two subfamilies differ primarily according to cranial depth and distal tooth row extension in the time-averaged comparison (where the probabilities are not quite significant), their differences are better attributed to the transverse width of the occipital region in the MAZ-1 comparison (where the probabilities are significant). It is possible that morphological disparity between centrosaurines and chasmosaurines may truly manifest itself differently within MAZ-1, but it is also likely that the smaller samples in this assemblage zone do not adequately capture the true ecological signal therein, and, in fact, artificially inflate statistical significance by reducing taxonomic overlap in morphospace [117].

There is some evidence, however, that ceratopsid subfamilies differ at least partly according to cranial depth in MAZ-1, as in the time-averaged analysis. This might be taken as tentative support for the finding of Henderson [44] that centrosaurines possess taller crania than chasmosaurines, making them more resistant to bending and torsional stresses. These differences were said to have facilitated niche partitioning between the two subfamilies, as centrosaurines presumably would have been capable of subsisting on a more resistant plant diet than sympatric chasmosaurines. Nonetheless, although Henderson [44] was careful to account for the confounding effects of taphonomic distortion, he considered only a single specimen per species, and therefore did not account for intraspecific variation. This omission is likely to have introduced some systematic bias into the results because numerous studies have shown that individual ceratopsid species actually vary quite widely, even when ontogenetic effects are accounted for [34], [109], [118]–[126]. For example, long-faced *Centrosaurus apertus* have been described (e.g., *Ce*. “*longirostris*” [127]), as well as short-faced *Chasmosaurus belli* (e.g., *Ch*. “*brevirostris*” [34]). It is therefore necessary that statistical approaches be taken to account for the significance of this variation.

#### Hadrosauridae

The inferred hadrosaurid ecomorph is characterized by a relatively large, narrow skull (though not as large as in ceratopsids), distally extended tooth row, and ventrally deflected snout. Hadrosaurids do not differ appreciably from ceratopsids in either the offset between the jaw joint and coronoid process apex or the distal extension of the tooth row, so it is likely that both jaw systems shared a similar mechanical advantage. However, the smaller size of the hadrosaurid skull suggests that these animals possessed a slightly weaker bite than ceratopsids.

Reconstruction of the hadrosaurid feeding envelope suggests that these animals could browse at heights up to 4 m above ground level [5], but it is otherwise unclear which height they browsed at most regularly. Unfortunately, those morphological features of the skull that correlate with feeding height do not clarify the matter. For example, the paroccipital processes are relatively narrow, a condition common among high-level browsers. However, the ventral deflection of the snout is most commonly observed among low-level grazers. Relative beak width is intermediate between that of ankylosaurs and ceratopsids, and does not otherwise provide convincing evidence for browse height. This unique combination of morphological characters might therefore indicate that hadrosaurids were equally comfortable browsing at both high and low levels. It is not difficult to imagine these animals feeding low in the herb layer, occasionally rearing up to feed bipedally among the surrounding shrubs when a herd of low-browsing ceratopsids passed through the area [5].

The strong jaws and large feeding heights of hadrosaurids suggests that these animals could subsist on a variety of plant types, and, as the largest members of the DPF megaherbivore assemblage, hadrosaurids likely possessed correspondingly large niche breadths [128]. Their dentition was probably capable of both crushing and shearing functions [43], [46], and could therefore process both tough and hard foodstuffs, encompassing a variety of browse types. Circumstantial evidence in favour of this hypothesis comes by way of fossil gut contents (enterolites), associated with various hadrosaurids, that contain conifer and angiosperm twigs and stems, bark, seeds, and leaves [129]–[131], although some have also cautioned that these materials may have been washed into the gut cavity post-mortem [130], [132]. Chin and Gill [133] and Chin [134] also reported on hadrosaurid coprolites containing an abundance of conifer wood, which cannot have been derived allochthonously.

Hadrosaurid subfamilies differ most noticeably in the development of cranial crests [135]–[137], but from the perspective of inferred dietary ecomorphology, hadrosaurine skulls are consistently larger than those of lambeosaurines. Hutchinson [138] noted that, in cases where two closely-related species, occupying the same position on the food-web, coexist, the skull of the larger form usually exceeds that of the smaller form in length by a ratio of ∼1.3, a figure that has come to be known as the Hutchinsonian ratio [139]. Although there is some question as to the actual statistical validity of this ratio [140]–[144], it is generally thought that these size differences are what allow closely related species to specialize on different foodstuffs, thereby circumventing interspecific competition. Interestingly, the ratio of mean skull length (measured as the distance from the jaw joint to the premaxillary apex) between hadrosaurines (754 mm) and lambeosaurines (612 mm) is 1.23, which is close to the figure of 1.3 noted by Hutchinson [138]. This difference in size could mean that the larger hadrosaurines incorporated less digestible plant matter in their diet than lambeosaurines. Although we found no other morphological characters to corroborate this hypothesis, it has been noted that hadrosaurines tend to exhibit more steeply inclined tooth facets than lambeosaurines, equating to a higher capacity for shearing in the former taxon [43], [46], [145]. This, in turn, would allow hadrosaurines to more effectively rend tougher, more fibrous plant tissues [82].

Numerous authors [37], [42], [43], [45] have argued that hadrosaurines generally possess relatively wider, squarer beaks than lambeosaurines, attributing this distinction to differences in their feeding ecologies. Unfortunately, previous attempts to quantify variation in hadrosaurid beak shape have not controlled for time-averaging, and have instead grouped forms spanning much of the Late Cretaceous. Thus, genera such as the late Campanian *Lambeosaurus* were compared alongside the late Maastrichtian *Edmontosaurus*, although the two were separated in time by ∼10 Ma. The time-constrained approach taken here suggests that sympatric hadrosaurines and lambeosaurines did not always differ in beak shape. Overlap in beak shape was noted by both Carrano et al. [43] and Whitlock [45], but the palaeoecological implications of this were not addressed. In fact, the DFA results suggest that, if anything, hadrosaurines possessed relatively narrower beaks than lambeosaurines within the boundaries of the DPF, but a posthoc Mann-Whitney U test reveals that the differences in arcsine-transformed relative beak width are not quite significant at *p*<0.05 (N = 33, U = 64, *p* = 0.079).

Chapman and Brett-Surman [146] also noted, on the basis of geometric morphometrics, that lambeosaurines have more ventrally deflected beaks than hadrosaurines. There is some support for this position, particularly in light of the results for the time-averaged analysis. This might be taken as evidence that lambeosaurines habitually fed closer to the ground than hadrosaurines. If so, this interpretation runs contrary to that of Carrano et al. [43], who suggested, on the basis of beak, tooth, and hindlimb morphology, that hadrosaurines foraged near to the ground in open habitats, whereas lambeosaurines foraged in closed habitats.

Finally, the results of Dodson’s [36] morphometric investigation of lambeosaurine skulls are supported here. His survey comprised 48 variables measured over 36 specimens, and the data were examined using bivariate and multivariate approaches. Dodson ([36]: p. 50) noted that “the differences among the five [morphological patterns among lambeosaurines] relate not to structures that have apparent significance in the differential utilization of trophic resources necessary for the coexistence of closely related species of large animals. Instead, they are confined to several parameters of the bony crest.” This finding was especially surprising to Dodson because it was then assumed that the DPF represented a single ‘snapshot’ in time, and that all dinosaurs from the DPF were contemporaneous. This hypothesis has since been falsified, and the temporal overlap of the lambeosaurines minimized [15], [17], [147], [148].

### Evolutionary Palaeoecology

**Figure 6:**
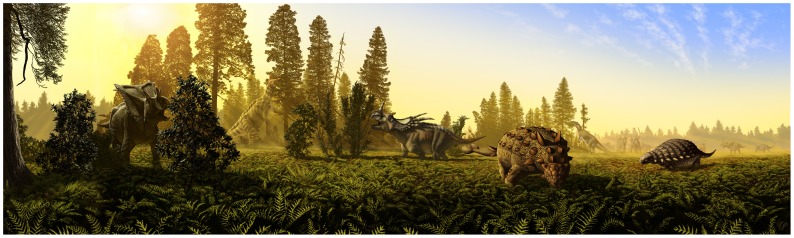
Depiction of dietary niche partitioning among megaherbivorous dinosaurs from the DPF (MAZ-2). Left to right: *Chasmosaurus belli*, *Lambeosaurus lambei*, *Styracosaurus albertensis*, *Euoplocephalus tutus*, *Prosaurolophus maximus*, *Panoplosaurus mirus*. A herd of *S. albertensis* looms in the background. Image courtesy of J.T. Csotonyi.

Of the 12 genera considered here–six of which typically coexisted at any given time [17]–five or six distinct ecomorphs are recovered using skull morphometrics. Ankylosaurs, ceratopsids, and hadrosaurids are each characterized by unique morphologies, the distinguishing characteristics being concentrated in the absolute size of the skull, and in its relative width, degree of ventral deflection of the snout, distal extension of the tooth row, and depth of the mandible. Ceratopsids and hadrosaurids are more alike than ankylosaurs, probably a reflection of the more recent common ancestry of the first two taxa (Figure 1, [149]). This implies that ankylosaurs were least likely to compete with the other megaherbivores from the DPF simply as a result of their more distant phylogenetic relatedness. Corroborating this hypothesis, the convergent evolution of dental batteries in ceratopsids and hadrosaurids suggests adaptation to the comminution of similar plant food (but see [114]). Ankylosaurids and nodosaurids are themselves distinguished primarily by differences in the construction of the mandible, and hadrosaurines and lambeosaurines differ mainly in skull size. Due to sample size limitations, it is difficult to determine whether centrosaurines and chasmosaurines differ at all, but there is reasonable evidence to suggest that they did. How they differ is also not immediately obvious, and the signal may change depending on whether time-averaging is minimized. Nonetheless, conventional knowledge that the two subfamilies differ according to cranial depth is tentatively confirmed. Therefore, with the above considerations in mind, the contention that dietary niche partitioning supported the rich megaherbivore diversity of the DPF is confirmed by this study (Figure 6).

There is no evidence for dietary niche partitioning among genera belonging to the same subfamily. For example, all hadrosaurine genera occupy nearly identical regions of morphospace, as do all genera within Lambeosaurinae, Centrosaurinae, and Chasmosaurinae. This is hardly surprising in light of the fact that consubfamilial taxa rarely lived in sympatry [17]. In fact, it may be precisely because of the morphological similarity of such taxa that they were unable to coexist, owing to the effects of competitive exclusion. In those rare instances where such taxa do overlap in time [17], their coexistence is either short-lived (e.g., ∼214 Ka for *Corythosaurus* and *Lambeosaurus*) or involves rare or transient forms (e.g., *Parasaurolophus*). The apparent rarity of the ankylosaurid *Dyoplosaurus* in MAZ-1 of the DPF, where *Euoplocephalus* is most common [17], also fits this pattern. These stable biostratigraphic patterns might be taken as evidence that the megaherbivorous dinosaur assemblage of the DPF was structured by the effects of competition, rather than having assembled via stochastic processes [150]. The rarity of sympatric consubfamilial taxa suggests that niche space was saturated and could not accommodate the addition of new species without the concomitant loss of already established competitors. This hypothesis further predicts that taxonomic overlap in morphospace should remain negligible with the recovery of additional fossils, and that the assemblage should adhere to certain ‘assembly rules’ [151] whereby the addition of new species results in an increase in total morphospace (niche) volume, an increase in morphological (niche) specificity, or the localized extinction of competitors [47]. Where taxonomic overlap in morphospace does occur, it should involve only rare taxa that would not have posed serious competition to more established members of the assemblage.

The temporal stability of the morphological patterns identified here (compare Figures 3–5), spanning ∼1.5 Ma, supports the contention of Brinkman et al. [152] that fossils from the upper Campanian Belly River Group (which includes the DPF) constitute a chronofauna. This is a term introduced by Olson ([153]: p. 181) to refer to “a geographically restricted, natural assemblage of interacting animal populations that has maintained its basic structure over a geologically significant period of time.” Thus, while individual species appear and disappear with time, the ecological relationships of the chronofauna remain stable. Olson [153] accounted for this stability with reference to environmental stasis, but the megaherbivore chronofauna of the DPF appears to have been rather impervious to environmental change, as the formation itself records the gradual transgression of the Western Interior Seaway [11], [12]. Perhaps the change was slow enough that the megaherbivore chronofauna could adapt accordingly, but there is no evidence to date that species turnover in the DPF responded to environmental change [17]. The apparent displacement of ankylosaurs from the upper limits of the DPF [17], [113], [154] may indicate a reshaping of the megaherbivore chronofauna in response to the encroaching sea, with ankylosaur congeners appearing in younger sediments of the Horseshoe Canyon Formation subsequent to the regression of the sea [52]. Alternatively, DiMichele et al. [155] have also invoked evolved mutualisms, historical contingency, and the ‘law of large numbers’ to account for ecological stasis in fossil assemblages.

### Conclusions

In their appraisal of research into dinosaur feeding behaviour, Barrett and Rayfield ([156]: p. 218) recently lamented the lack of studies attempting to “place feeding within more holistic evolutionary or ecological frameworks”. The present study is an attempt to address this concern by examining dinosaur feeding in a geographically and temporally constrained manner, thereby approximating true ecological relationships. The implementation of statistical procedures also allows for more robust comparisons and provides a means by which to gauge the palaeoecological significance of variation.

This study supports the hypothesis that the great standing crop megaherbivore diversity of the DPF (and by extrapolation, much of Laramidia) is largely attributable to dietary niche partitioning. Coexisting ankylosaurids and nodosaurids, hadrosaurines and lambeosaurines, and probably centrosaurines and chasmosaurines, differ significantly in their morphologies, and likely differed in their food preferences as a result. The interpretation that niche partitioning facilitated megaherbivore coexistence in the DPF can be tested further by examining the response of the assemblage structure to the appearance of new species as new fossil discoveries are made, and by examining other ecological proxies including dental microwear and stable isotopes. The inferred ecological relationships appear to have been stable over the ∼1.5 million year span of the DPF, as revealed by time-constrained analyses of morphological patterns. This stability is characteristic of chronofaunas [153].

Variation in such aspects as body size, beak breadth, jaw mechanics, and tooth morphology are commonly cited as evidence for dinosaur feeding ecology [9], [37]–[46], [69], [70], [157]–[164], but this study identifies several other morphological variables that may help to reveal subtle differences in dinosaur palaeoecology. Chief among these are variables relating to the development of the nuchal musculature and the ventral deflection of the beak. Unfortunately, the functional significance of these and other morphological variables is only poorly understood, emphasizing the need for further detailed analyses of herbivore functional morphology and the development of general functional principles [165].

## Supporting Information

Information S1 Supporting ordination data (eigenvalues and variable loadings) for the results of this study.
(DOCX)

Table S1 Raw data used in this study.
(DOCX)
