# Supplementary material for: Skull Ecomorphology of Megaherbivorous Dinosaurs from the Dinosaur Park Formation (Upper Campanian) of Alberta, Canada
Source: PLoS One. 2013 Jul 10;8(7):e67182. doi: 10.1371/journal.pone.0067182 (PMC3707905; doi:10.1371/journal.pone.0067182)
Supplement: Information S1 — Supporting ordination data (eigenvalues and variable loadings) for the results of this study. (DOCX) [file pone.0067182.s001.docx]

Supporting Information S2. Supporting ordination data (eigenvalues and variable loadings) for the results of this study.

Table S2. Results for the time-averaged, suborder/family level PCA.

| PC | Eigenvalue | % variance | Cumulative % |
| --- | --- | --- | --- |
| 1 | 0.186643 | 52.183 | 52.183 |
| 2 | 0.108687 | 30.388 | 82.571 |
| 3 | 0.021298 | 5.9546 | 88.5256 |
| 4 | 0.014996 | 4.1928 | 92.7184 |
| 5 | 0.007147 | 1.9982 | 94.7166 |
| 6 | 0.006985 | 1.9529 | 96.6695 |
| 7 | 0.00433 | 1.2105 | 97.88 |
| 8 | 0.003362 | 0.94005 | 98.82005 |
| 9 | 0.002154 | 0.60229 | 99.42234 |
| 10 | 0.001209 | 0.33793 | 99.76027 |
| 11 | 0.000591 | 0.1652 | 99.92547 |
| 12 | 0.000267 | 0.074591 | 100 |

Table S3. Variable loadings for the time-averaged, suborder/family level PCA.

|  | PC | | | | | | | | | | | |
| --- | --- | --- | --- | --- | --- | --- | --- | --- | --- | --- | --- | --- |
| Variable | 1 | 2 | 3 | 4 | 5 | 6 | 7 | 8 | 9 | 10 | 11 | 12 |
| 1 | 0.3207 | 0.1384 | 0.0047 | -0.0672 | 0.0565 | 0.0350 | 0.1986 | 0.2281 | 0.1433 | -0.2398 | -0.0334 | 0.8357 |
| 2 | 0.3572 | 0.1774 | -0.0224 | -0.0972 | 0.0104 | 0.0340 | 0.3014 | 0.2214 | 0.1250 | -0.4659 | -0.4739 | -0.4824 |
| 3 | 0.2777 | 0.0664 | 0.0772 | -0.1563 | 0.0604 | 0.0764 | 0.1535 | 0.1164 | 0.1080 | -0.1713 | 0.8621 | -0.2394 |
| 4 | 0.0540 | -0.2302 | 0.6145 | -0.6382 | 0.2113 | -0.2220 | -0.1317 | -0.0064 | -0.1869 | 0.0021 | -0.1122 | 0.0188 |
| 5 | -0.0025 | -0.2193 | 0.3346 | 0.1670 | 0.1538 | 0.8734 | -0.0193 | -0.1158 | -0.0084 | -0.0866 | -0.0639 | 0.0122 |
| 6 | 0.1591 | 0.1611 | 0.1251 | 0.0397 | 0.2848 | 0.0623 | 0.2156 | 0.3783 | 0.2598 | 0.7567 | -0.0905 | -0.0927 |
| 7 | -0.0472 | 0.5280 | 0.3224 | 0.3481 | 0.5015 | -0.2141 | -0.0167 | -0.4161 | 0.0424 | -0.1447 | 0.0181 | 0.0014 |
| 8 | 0.4059 | 0.0152 | -0.0196 | -0.0249 | -0.1050 | 0.0081 | -0.7213 | -0.1225 | 0.5318 | 0.0285 | -0.0534 | -0.0337 |
| 9 | 0.4625 | 0.0520 | -0.0869 | -0.1797 | -0.2521 | 0.0680 | 0.3311 | -0.6751 | -0.1424 | 0.2941 | -0.0502 | 0.0265 |
| 10 | 0.3857 | -0.6314 | 0.1301 | 0.5427 | 0.1227 | -0.3297 | 0.0941 | 0.0253 | -0.0820 | -0.0278 | 0.0114 | -0.0178 |
| 11 | 0.3668 | 0.2734 | -0.1347 | 0.0834 | 0.0956 | 0.1134 | -0.3776 | 0.2430 | -0.7323 | 0.0731 | 0.0065 | -0.0196 |
| 12 | -0.0078 | 0.2506 | 0.5842 | 0.2634 | -0.7024 | -0.0610 | 0.0193 | 0.1588 | -0.0422 | 0.0396 | 0.0218 | 0.0010 |

Table S4. Variable loadings for the time-averaged, suborder/family level DFA.

|  | DF | |
| --- | --- | --- |
| PC | 1 | 2 |
| 1 | 3.6002 | -0.81702 |
| 2 | 1.4234 | 1.925 |
| 3 | -0.9215 | -0.12294 |
| 4 | 0.39485 | 0.60565 |
| 5 | -0.41136 | 0.37414 |
| 6 | -0.00953 | -0.28906 |

Table S5. Results for the time-averaged, ankylosaur family PCA.

| PC | Eigenvalue | % variance | Cumulative % |
| --- | --- | --- | --- |
| 1 | 0.069201 | 54.734 | 54.734 |
| 2 | 0.033777 | 26.716 | 81.45 |
| 3 | 0.010242 | 8.1003 | 89.5503 |
| 4 | 0.004245 | 3.3572 | 92.9075 |
| 5 | 0.003042 | 2.4063 | 95.3138 |
| 6 | 0.00224 | 1.7714 | 97.0852 |
| 7 | 0.001526 | 1.2067 | 98.2919 |
| 8 | 0.000838 | 0.66254 | 98.95444 |
| 9 | 0.000579 | 0.45785 | 99.41229 |
| 10 | 0.000413 | 0.32672 | 99.73901 |
| 11 | 0.000281 | 0.22224 | 99.96125 |
| 12 | 4.97x10^-5^ | 0.039345 | 100 |

Table S6. Variable loadings for the time-averaged, ankylosaur family PCA.

|  | PC | | | | | | | | | | | |
| --- | --- | --- | --- | --- | --- | --- | --- | --- | --- | --- | --- | --- |
| Variable | 1 | 2 | 3 | 4 | 5 | 6 | 7 | 8 | 9 | 10 | 11 | 12 |
| 1 | 0.1605 | 0.1830 | 0.1732 | 0.1197 | 0.0245 | 0.3076 | -0.2022 | -0.2367 | 0.2106 | 0.3511 | -0.4308 | 0.5925 |
| 2 | 0.1671 | 0.2322 | 0.1905 | 0.0412 | -0.1522 | 0.3498 | -0.1283 | -0.3196 | 0.5502 | -0.2101 | 0.4352 | -0.2824 |
| 3 | 0.1582 | 0.3063 | 0.1980 | -0.0073 | 0.3110 | 0.2876 | 0.0566 | -0.0473 | -0.2883 | -0.2754 | -0.5250 | -0.4716 |
| 4 | 0.2501 | 0.2870 | 0.3852 | -0.1790 | -0.2277 | -0.6264 | -0.1389 | -0.3727 | -0.2550 | -0.0456 | 0.0450 | 0.0559 |
| 5 | -0.1424 | 0.0853 | 0.3090 | -0.5459 | 0.6449 | -0.0043 | -0.1034 | 0.1538 | 0.0945 | 0.2151 | 0.2632 | 0.0643 |
| 6 | -0.1317 | 0.3818 | 0.1007 | -0.1090 | -0.2886 | -0.2071 | -0.1186 | 0.6426 | 0.4029 | -0.1616 | -0.2707 | 0.0019 |
| 7 | -0.0679 | 0.1981 | 0.0461 | -0.2063 | -0.0406 | 0.1299 | 0.7278 | -0.0743 | -0.0189 | -0.4007 | 0.0753 | 0.4372 |
| 8 | 0.0098 | 0.4261 | -0.4036 | 0.2878 | 0.3633 | -0.3803 | 0.2999 | -0.1735 | 0.2963 | 0.2617 | -0.0257 | -0.1380 |
| 9 | 0.8363 | -0.0137 | 0.0251 | 0.1183 | 0.0755 | 0.0356 | 0.1487 | 0.4336 | -0.0755 | 0.1071 | 0.2161 | 0.0693 |
| 10 | -0.2482 | -0.1320 | 0.6779 | 0.5743 | 0.0627 | -0.0839 | 0.2737 | 0.1228 | 0.0238 | 0.1440 | 0.0736 | -0.0630 |
| 11 | -0.2106 | 0.5164 | -0.1230 | 0.3371 | 0.0724 | 0.1687 | -0.3229 | 0.1536 | -0.4371 | -0.1250 | 0.3745 | 0.2277 |
| 12 | -0.1049 | 0.2798 | 0.0150 | -0.2433 | -0.4242 | 0.2583 | 0.2729 | 0.0430 | -0.2193 | 0.6368 | 0.0782 | -0.2619 |

Table S7. Results for the time-averaged, ceratopsid subfamily PCA.

| PC | Eigenvalue | % variance | Cumulative % variance |
| --- | --- | --- | --- |
| 1 | 0.225234 | 75.139 | 75.139 |
| 2 | 0.046105 | 15.381 | 90.52 |
| 3 | 0.011846 | 3.9519 | 94.4719 |
| 4 | 0.005669 | 1.8912 | 96.3631 |
| 5 | 0.003683 | 1.2285 | 97.5916 |
| 6 | 0.003187 | 1.0632 | 98.6548 |
| 7 | 0.001558 | 0.51979 | 99.17459 |
| 8 | 0.001141 | 0.3807 | 99.55529 |
| 9 | 0.000712 | 0.23747 | 99.79276 |
| 10 | 0.000414 | 0.13795 | 99.93071 |
| 11 | 0.000159 | 0.053093 | 99.9838 |
| 12 | 5.02x10^-5^ | 0.01676 | 100 |

Table S8. Variable loadings for the time-averaged, ceratopsid subfamily PCA.

|  | PC | | | | | | | | | | | |
| --- | --- | --- | --- | --- | --- | --- | --- | --- | --- | --- | --- | --- |
| Variable | 1 | 2 | 3 | 4 | 5 | 6 | 7 | 8 | 9 | 10 | 11 | 12 |
| 1 | -0.0333 | 0.1781 | 0.0160 | -0.0015 | -0.0187 | 0.1251 | 0.0820 | 0.0475 | 0.0647 | 0.1376 | 0.9103 | 0.3003 |
| 2 | -0.0369 | 0.1763 | 0.0443 | -0.0143 | -0.0825 | 0.3007 | 0.1438 | 0.2358 | 0.1682 | 0.0793 | 0.1537 | -0.8565 |
| 3 | 0.0418 | 0.1743 | 0.1179 | 0.2089 | -0.1543 | 0.3609 | 0.0005 | 0.3778 | -0.6156 | 0.4166 | -0.1813 | 0.1675 |
| 4 | 0.0368 | 0.6531 | 0.4645 | -0.4760 | 0.2042 | -0.2015 | -0.0326 | -0.1624 | -0.1020 | 0.0416 | -0.0885 | -0.0078 |
| 5 | 0.0533 | 0.0316 | -0.2819 | 0.1373 | 0.8496 | -0.0908 | 0.2140 | 0.1430 | 0.0442 | 0.3125 | -0.0394 | -0.0206 |
| 6 | -0.0376 | 0.1445 | -0.0123 | -0.0698 | 0.1853 | 0.2740 | 0.2274 | 0.4445 | -0.0502 | -0.7589 | -0.0488 | 0.1820 |
| 7 | -0.0468 | 0.2561 | -0.2665 | 0.1698 | -0.0743 | 0.2809 | 0.5208 | -0.6555 | -0.1840 | -0.0895 | -0.0710 | -0.0063 |
| 8 | 0.2214 | 0.1022 | 0.1016 | 0.1451 | -0.3265 | -0.4625 | 0.6372 | 0.2894 | 0.2460 | 0.1242 | -0.1021 | 0.1116 |
| 9 | -0.0122 | 0.0962 | 0.1325 | -0.0398 | -0.0109 | 0.5364 | -0.0363 | -0.0175 | 0.6721 | 0.2382 | -0.2776 | 0.3105 |
| 10 | 0.9649 | 0.0093 | -0.1095 | -0.0570 | 0.0044 | 0.1400 | -0.1321 | -0.0712 | -0.0166 | -0.0803 | 0.0576 | -0.0378 |
| 11 | 0.0591 | 0.0989 | 0.5272 | 0.7658 | 0.1682 | -0.0490 | -0.1525 | -0.1423 | 0.0742 | -0.1911 | 0.0496 | -0.0601 |
| 12 | -0.0655 | 0.6026 | -0.5474 | 0.2543 | -0.1749 | -0.1926 | -0.3933 | 0.1264 | 0.1474 | -0.0522 | -0.0691 | 0.0240 |

Table S9. Results for the time-averaged, hadrosaurid subfamily PCA.

| PC | Eigenvalue | % variance | Cumulative % variance |
| --- | --- | --- | --- |
| 1 | 0.072896 | 46.168 | 46.168 |
| 2 | 0.057289 | 36.283 | 82.451 |
| 3 | 0.012217 | 7.7372 | 90.1882 |
| 4 | 0.00477 | 3.0212 | 93.2094 |
| 5 | 0.004074 | 2.5803 | 95.7897 |
| 6 | 0.002268 | 1.4363 | 97.226 |
| 7 | 0.001491 | 0.94431 | 98.17031 |
| 8 | 0.001104 | 0.69911 | 98.86942 |
| 9 | 0.000785 | 0.49715 | 99.36657 |
| 10 | 0.000693 | 0.43909 | 99.80566 |
| 11 | 0.000226 | 0.14332 | 99.94898 |
| 12 | 8.03x10^-5^ | 0.050859 | 100 |

Table S10. Variable loadings for the time-averaged, hadrosaurid subfamily PCA.

|  | PC | | | | | | | | | | | |
| --- | --- | --- | --- | --- | --- | --- | --- | --- | --- | --- | --- | --- |
| Variable | 1 | 2 | 3 | 4 | 5 | 6 | 7 | 8 | 9 | 10 | 11 | 12 |
| 1 | 0.2192 | 0.0404 | 0.3219 | 0.0887 | 0.0419 | 0.1742 | -0.1525 | -0.3984 | 0.0974 | 0.1441 | -0.1442 | -0.7574 |
| 2 | 0.2039 | 0.0741 | 0.3243 | 0.1074 | 0.0714 | 0.0833 | -0.0886 | -0.5328 | 0.0925 | -0.0059 | 0.5784 | 0.4351 |
| 3 | 0.1986 | 0.0650 | 0.3195 | 0.0702 | 0.0077 | 0.0461 | -0.3072 | 0.0410 | 0.1659 | 0.2909 | -0.6553 | 0.4578 |
| 4 | 0.2492 | 0.1217 | 0.3217 | -0.6399 | 0.4243 | -0.3686 | 0.0513 | 0.0956 | -0.0790 | -0.2717 | -0.0301 | -0.0377 |
| 5 | 0.3784 | 0.0911 | -0.4617 | 0.4380 | 0.6447 | -0.0867 | -0.0892 | -0.0181 | -0.0696 | -0.0630 | -0.0562 | 0.0024 |
| 6 | 0.2197 | 0.0646 | 0.3126 | 0.1934 | -0.0944 | 0.3096 | -0.1799 | 0.3916 | -0.6993 | -0.1267 | 0.1287 | -0.0197 |
| 7 | 0.4668 | 0.3220 | -0.1110 | 0.1634 | -0.5905 | -0.4907 | 0.0492 | -0.0413 | 0.0295 | -0.2047 | -0.0397 | -0.0371 |
| 8 | 0.1340 | 0.1064 | 0.1098 | 0.0502 | 0.0711 | -0.1138 | 0.6869 | 0.0133 | -0.2273 | 0.6431 | 0.0225 | 0.0183 |
| 9 | 0.1360 | 0.0894 | 0.1363 | 0.0857 | 0.0509 | -0.0507 | -0.1861 | 0.6107 | 0.5252 | 0.2739 | 0.4062 | -0.1383 |
| 10 | 0.5645 | -0.7082 | -0.2038 | -0.2606 | -0.1540 | 0.1870 | 0.0432 | 0.0176 | 0.0434 | 0.0714 | 0.0347 | 0.0376 |
| 11 | 0.1270 | 0.0835 | 0.1958 | 0.1835 | 0.0431 | 0.4218 | 0.5615 | 0.1271 | 0.3521 | -0.4919 | -0.1593 | 0.0474 |
| 12 | 0.1681 | 0.5744 | -0.3897 | -0.4459 | -0.0808 | 0.5021 | -0.0738 | -0.0405 | 0.0151 | 0.1521 | 0.0314 | 0.0336 |

Table S11. Results for the time-averaged, hadrosaurid genus PCA.

| PC | Eigenvalue | % variance | Cumulative % variance |
| --- | --- | --- | --- |
| 1 | 0.0732 | 46.304 | 46.304 |
| 2 | 0.056716 | 35.877 | 82.181 |
| 3 | 0.012434 | 7.8653 | 90.0463 |
| 4 | 0.00485 | 3.0679 | 93.1142 |
| 5 | 0.004171 | 2.6386 | 95.7528 |
| 6 | 0.002289 | 1.448 | 97.2008 |
| 7 | 0.001512 | 0.95657 | 98.15737 |
| 8 | 0.001132 | 0.71602 | 98.87339 |
| 9 | 0.000767 | 0.48516 | 99.35855 |
| 10 | 0.000702 | 0.44386 | 99.80241 |
| 11 | 0.000231 | 0.14595 | 99.94836 |
| 12 | 8.17x10^-5^ | 0.051691 | 100 |

Table S12. Variable loadings for the time-averaged, hadrosaurid genus PCA.

|  | PC | | | | | | | | | | | |
| --- | --- | --- | --- | --- | --- | --- | --- | --- | --- | --- | --- | --- |
| Variable | 1 | 2 | 3 | 4 | 5 | 6 | 7 | 8 | 9 | 10 | 11 | 12 |
| 1 | 0.2275 | -0.0017 | 0.3178 | 0.0830 | 0.0459 | 0.1613 | -0.1678 | -0.3933 | 0.1557 | 0.1000 | -0.1435 | -0.7580 |
| 2 | 0.2171 | 0.0332 | 0.3211 | 0.1032 | 0.0743 | 0.0684 | -0.1084 | -0.5248 | 0.1271 | -0.0571 | 0.5755 | 0.4359 |
| 3 | 0.2088 | 0.0285 | 0.3187 | 0.0724 | 0.0089 | 0.0506 | -0.3063 | 0.0566 | 0.2214 | 0.2527 | -0.6541 | 0.4565 |
| 4 | 0.2625 | 0.0852 | 0.3278 | -0.6281 | 0.4147 | -0.3898 | 0.0430 | 0.1118 | -0.1669 | -0.2314 | -0.0292 | -0.0373 |
| 5 | 0.3841 | 0.0498 | -0.4656 | 0.4372 | 0.6429 | -0.0907 | -0.0887 | -0.0153 | -0.0869 | -0.0419 | -0.0558 | 0.0022 |
| 6 | 0.2278 | 0.0277 | 0.3126 | 0.1983 | -0.0901 | 0.3520 | -0.1385 | 0.3370 | -0.7233 | 0.0635 | 0.1343 | -0.0194 |
| 7 | 0.5050 | 0.2550 | -0.1065 | 0.1759 | -0.6011 | -0.4788 | 0.0417 | -0.0208 | -0.0290 | -0.2029 | -0.0402 | -0.0363 |
| 8 | 0.1474 | 0.0862 | 0.1114 | 0.0518 | 0.0702 | -0.1127 | 0.7003 | -0.0433 | -0.0429 | 0.6655 | 0.0265 | 0.0181 |
| 9 | 0.1475 | 0.0670 | 0.1364 | 0.0893 | 0.0503 | -0.0360 | -0.1690 | 0.6470 | 0.5356 | 0.1595 | 0.4061 | -0.1377 |
| 10 | 0.4616 | -0.7694 | -0.2291 | -0.2759 | -0.1479 | 0.1808 | 0.0438 | 0.0178 | 0.0580 | 0.0599 | 0.0340 | 0.0380 |
| 11 | 0.1408 | 0.0583 | 0.1891 | 0.1620 | 0.0552 | 0.4111 | 0.5560 | 0.1270 | 0.2353 | -0.5754 | -0.1701 | 0.0457 |
| 12 | 0.2405 | 0.5615 | -0.3766 | -0.4563 | -0.0724 | 0.4877 | -0.0744 | -0.0552 | 0.0614 | 0.1393 | 0.0315 | 0.0326 |

Table S13. Variable loadings for the time-averaged, hadrosaurid genus DFA.

|  | DF | | |
| --- | --- | --- | --- |
| PC | 1 | 2 | 3 |
| 1 | 0.77489 | -0.18685 | -0.37033 |
| 2 | 0.21329 | 0.22028 | 0.60709 |
| 3 | 0.95579 | 0.51117 | 0.1243 |
| 4 | 0.030212 | -0.35577 | 0.67517 |
| 5 | -0.51443 | 0.73872 | -0.03555 |

Table S14. Results for the MAZ-1, suborder/family level PCA.

| PC | Eigenvalue | % variance | Cumulative % variance |
| --- | --- | --- | --- |
| 1 | 0.15028 | 46.223 | 46.223 |
| 2 | 0.116148 | 35.725 | 81.948 |
| 3 | 0.021347 | 6.5659 | 88.5139 |
| 4 | 0.013845 | 4.2583 | 92.7722 |
| 5 | 0.007778 | 2.3924 | 95.1646 |
| 6 | 0.00724 | 2.2269 | 97.3915 |
| 7 | 0.003314 | 1.0193 | 98.4108 |
| 8 | 0.002437 | 0.74948 | 99.16028 |
| 9 | 0.001548 | 0.47608 | 99.63636 |
| 10 | 0.000782 | 0.24055 | 99.87691 |
| 11 | 0.000321 | 0.098723 | 99.97563 |
| 12 | 8.07x10^-5^ | 0.024834 | 100 |

Table S15. Variable loadings for the MAZ-1, suborder/family level PCA.

|  | PC | | | | | | | | | | | |
| --- | --- | --- | --- | --- | --- | --- | --- | --- | --- | --- | --- | --- |
| Variable | 1 | 2 | 3 | 4 | 5 | 6 | 7 | 8 | 9 | 10 | 11 | 12 |
| 1 | 0.2544 | 0.2232 | -0.0463 | 0.0363 | -0.0336 | 0.0403 | 0.3752 | -0.0652 | -0.0747 | -0.2498 | -0.1964 | 0.7923 |
| 2 | 0.2836 | 0.2493 | -0.0855 | 0.0637 | -0.0931 | 0.0682 | 0.3386 | -0.1151 | -0.1360 | -0.4363 | -0.3821 | -0.5916 |
| 3 | 0.2621 | 0.1369 | -0.0032 | 0.1868 | -0.0021 | 0.0330 | 0.2823 | -0.1434 | -0.1657 | -0.0365 | 0.8596 | -0.0927 |
| 4 | 0.1159 | -0.1968 | 0.3767 | 0.7877 | 0.1930 | -0.2625 | 0.0261 | 0.1073 | 0.2266 | -0.0554 | -0.1128 | -0.0019 |
| 5 | 0.1348 | -0.1694 | 0.3635 | -0.0399 | 0.2875 | 0.8392 | -0.0727 | 0.1244 | -0.0306 | -0.1038 | -0.0006 | 0.0060 |
| 6 | 0.1403 | 0.2382 | 0.0320 | -0.0087 | 0.2080 | 0.0500 | 0.4385 | 0.1639 | -0.0347 | 0.7835 | -0.1809 | -0.0988 |
| 7 | -0.1424 | 0.5366 | 0.2601 | -0.1847 | 0.6667 | -0.2317 | -0.1918 | -0.1631 | -0.0109 | -0.1643 | 0.0354 | -0.0084 |
| 8 | 0.3630 | 0.1639 | 0.0256 | 0.1062 | -0.1028 | -0.1124 | -0.4718 | 0.4024 | -0.6431 | 0.0643 | -0.0508 | 0.0389 |
| 9 | 0.3800 | 0.0847 | -0.0430 | 0.1356 | -0.1016 | 0.1297 | -0.3919 | -0.7338 | 0.1182 | 0.2823 | -0.1199 | 0.0301 |
| 10 | 0.5784 | -0.4605 | 0.1902 | -0.4908 | 0.1785 | -0.3496 | 0.0749 | 0.0169 | 0.1166 | -0.0428 | 0.0138 | -0.0302 |
| 11 | 0.3157 | 0.3726 | -0.2456 | -0.0172 | -0.0778 | 0.0920 | -0.2161 | 0.4252 | 0.6655 | -0.0610 | 0.1090 | -0.0200 |
| 12 | -0.0657 | 0.2688 | 0.7408 | -0.1808 | -0.5690 | -0.0570 | 0.0373 | -0.0033 | 0.1039 | 0.0417 | 0.0277 | -0.0124 |

Table S16. Variable loadings for the MAZ-1, suborder/family level DFA.

|  | DF | |
| --- | --- | --- |
| PC | 1 | 2 |
| 1 | 3.3679 | -1.6196 |
| 2 | 2.9917 | 1.6046 |
| 3 | -1.1139 | -0.17758 |
| 4 | -0.96599 | -0.62823 |
| 5 | -0.73891 | 0.20387 |

Table S17. Results for the MAZ-1, ceratopsid subfamily PCA.

| PC | Eigenvalue | % variance | Cumulative % variance |
| --- | --- | --- | --- |
| 1 | 0.10992 | 54.316 | 54.316 |
| 2 | 0.065596 | 32.414 | 86.73 |
| 3 | 0.014876 | 7.3511 | 94.0811 |
| 4 | 0.005124 | 2.5318 | 96.6129 |
| 5 | 0.003016 | 1.4902 | 98.1031 |
| 6 | 0.002533 | 1.2518 | 99.3549 |
| 7 | 0.000769 | 0.3802 | 99.7351 |
| 8 | 0.00036 | 0.17793 | 99.91303 |
| 9 | 0.000136 | 0.067344 | 99.98037 |
| 10 | 2.72x10^-5^ | 0.013431 | 99.99381 |
| 11 | 1.3210^-5^ | 0.006521 | 100 |

Table S18. Variable loadings for the MAZ-1, ceratopsid subfamily PCA.

|  | PC | | | | | | | | | | | |
| --- | --- | --- | --- | --- | --- | --- | --- | --- | --- | --- | --- | --- |
| Variable | 1 | 2 | 3 | 4 | 5 | 6 | 7 | 8 | 9 | 10 | 11 | 12 |
| 1 | 0.1191 | 0.0390 | 0.0287 | 0.0750 | 0.3042 | 0.2671 | 0.1806 | 0.0816 | 0.2714 | -0.7789 | 0.2581 | 0.1664 |
| 2 | 0.1327 | 0.0033 | 0.0523 | 0.0517 | 0.3233 | 0.3181 | 0.1932 | -0.1102 | -0.3820 | 0.1862 | -0.3476 | 0.6484 |
| 3 | 0.1267 | 0.0327 | 0.0909 | -0.0870 | 0.2135 | 0.1263 | 0.4243 | -0.4281 | -0.0627 | 0.3384 | 0.6210 | -0.1953 |
| 4 | 0.3005 | 0.2408 | 0.5532 | 0.1312 | 0.1312 | 0.2167 | -0.6409 | 0.0689 | -0.0437 | 0.1097 | 0.1806 | -0.0472 |
| 5 | -0.0047 | 0.1262 | -0.4107 | 0.7511 | -0.2992 | 0.1525 | -0.0694 | 0.0247 | -0.1915 | 0.0364 | 0.2898 | 0.1050 |
| 6 | 0.0984 | 0.0171 | -0.0917 | 0.2907 | 0.1150 | 0.3273 | 0.0051 | -0.3855 | 0.5741 | 0.1517 | -0.4512 | -0.2682 |
| 7 | 0.4309 | 0.0394 | -0.2640 | -0.1001 | 0.0706 | -0.1892 | 0.0140 | 0.3991 | 0.4998 | 0.3629 | 0.1762 | 0.3468 |
| 8 | 0.1010 | 0.2628 | 0.3587 | -0.0761 | -0.6126 | 0.3566 | 0.4408 | 0.2703 | 0.0757 | 0.0398 | -0.0937 | -0.0168 |
| 9 | -0.0302 | 0.0276 | 0.0424 | 0.2779 | 0.4729 | -0.0061 | 0.2768 | 0.5901 | -0.1792 | 0.1297 | -0.1367 | -0.4507 |
| 10 | -0.2541 | 0.9101 | -0.1219 | -0.0860 | 0.1553 | -0.2031 | 0.0260 | -0.1011 | 0.0395 | -0.0140 | -0.0606 | 0.0562 |
| 11 | 0.0781 | -0.0530 | 0.4896 | 0.4583 | -0.0276 | -0.6281 | 0.2455 | -0.1722 | 0.1247 | -0.0569 | -0.0696 | 0.1803 |
| 12 | 0.7647 | 0.1440 | -0.2242 | -0.0885 | -0.0928 | -0.1881 | 0.0472 | -0.1532 | -0.3218 | -0.2328 | -0.1992 | -0.2632 |

Table S19. Results for the MAZ-1, hadrosaurid subfamily PCA.

| PC | Eigenvalue | % variance | Cumulative % variance |
| --- | --- | --- | --- |
| 1 | 0.049433 | 45.981 | 45.981 |
| 2 | 0.034048 | 31.67 | 77.651 |
| 3 | 0.009242 | 8.5968 | 86.2478 |
| 4 | 0.004774 | 4.4406 | 90.6884 |
| 5 | 0.004325 | 4.023 | 94.7114 |
| 6 | 0.002498 | 2.3239 | 97.0353 |
| 7 | 0.001119 | 1.0409 | 98.0762 |
| 8 | 0.000896 | 0.83303 | 98.90923 |
| 9 | 0.000582 | 0.54149 | 99.45072 |
| 10 | 0.000446 | 0.41459 | 99.86531 |
| 11 | 0.00011 | 0.10248 | 99.96779 |
| 12 | 3.50x10^-5^ | 0.032511 | 100 |

Table S20. Variable loadings for the MAZ-1, hadrosaurid subfamily PCA.

|  | PC | | | | | | | | | | | |
| --- | --- | --- | --- | --- | --- | --- | --- | --- | --- | --- | --- | --- |
| Variable | 1 | 2 | 3 | 4 | 5 | 6 | 7 | 8 | 9 | 10 | 11 | 12 |
| 1 | 0.1386 | 0.2919 | 0.1780 | 0.1258 | -0.0503 | 0.0571 | -0.3403 | -0.2777 | 0.1758 | 0.1731 | -0.1271 | -0.7566 |
| 2 | 0.1233 | 0.2713 | 0.2094 | 0.0897 | -0.1519 | -0.0331 | -0.3736 | -0.2083 | 0.0535 | 0.2065 | 0.6743 | 0.3899 |
| 3 | 0.1587 | 0.2678 | 0.2183 | 0.0909 | 0.0370 | 0.1149 | 0.0721 | -0.3276 | 0.4818 | -0.1856 | -0.5038 | 0.4470 |
| 4 | 0.1505 | 0.1040 | 0.2927 | 0.4149 | 0.4590 | -0.6096 | 0.1007 | 0.1280 | -0.2247 | 0.1862 | -0.1125 | 0.0460 |
| 5 | 0.4816 | 0.1141 | -0.4520 | 0.3728 | -0.5579 | -0.2271 | 0.0716 | 0.0720 | -0.0830 | -0.1675 | -0.0614 | 0.0020 |
| 6 | 0.2270 | 0.1782 | 0.2155 | 0.0270 | 0.2517 | 0.1854 | -0.0830 | 0.1919 | -0.1522 | -0.7989 | 0.2252 | -0.1263 |
| 7 | 0.6323 | -0.0238 | 0.1100 | -0.7107 | 0.0250 | -0.1461 | 0.0473 | -0.0658 | -0.1668 | 0.1414 | -0.0779 | 0.0111 |
| 8 | 0.1448 | 0.0184 | 0.2466 | -0.0125 | -0.1372 | 0.0557 | 0.0933 | 0.7461 | 0.5358 | 0.1813 | 0.0824 | -0.0609 |
| 9 | 0.1525 | 0.0415 | 0.0843 | 0.1492 | 0.0570 | 0.2413 | 0.7983 | -0.2831 | 0.0538 | 0.1182 | 0.3473 | -0.1710 |
| 10 | 0.0835 | 0.5061 | -0.6107 | -0.0566 | 0.4898 | 0.2106 | -0.0419 | 0.1613 | 0.0597 | 0.2032 | 0.0259 | 0.0564 |
| 11 | 0.1382 | 0.0622 | 0.2525 | 0.2241 | -0.1254 | 0.5951 | -0.0635 | 0.1869 | -0.5327 | 0.2834 | -0.2631 | 0.1445 |
| 12 | 0.4053 | -0.6717 | -0.1541 | 0.2741 | 0.3309 | 0.2089 | -0.2515 | -0.1083 | 0.2203 | 0.0598 | 0.0698 | 0.0242 |

Table S21. Results for the MAZ-1, hadrosaurid genus PCA.

| PC | Eigenvalue | % variance | Cumulative % variance |
| --- | --- | --- | --- |
| 1 | 0.051788 | 47.492 | 47.492 |
| 2 | 0.033068 | 30.325 | 77.817 |
| 3 | 0.00926 | 8.4916 | 86.3086 |
| 4 | 0.005016 | 4.5998 | 90.9084 |
| 5 | 0.004143 | 3.7998 | 94.7082 |
| 6 | 0.002578 | 2.3644 | 97.0726 |
| 7 | 0.001154 | 1.0583 | 98.1309 |
| 8 | 0.00087 | 0.79815 | 98.92905 |
| 9 | 0.0006 | 0.55062 | 99.47967 |
| 10 | 0.000428 | 0.39288 | 99.87255 |
| 11 | 0.000105 | 0.096682 | 99.96923 |
| 12 | 3.41x10^-5^ | 0.031317 | 100 |

Table S22. Variable loadings for the MAZ-1, hadrosaurid genus PCA.

|  | PC | | | | | | | | | | | |
| --- | --- | --- | --- | --- | --- | --- | --- | --- | --- | --- | --- | --- |
| Variable | 1 | 2 | 3 | 4 | 5 | 6 | 7 | 8 | 9 | 10 | 11 | 12 |
| 1 | 0.1291 | 0.3271 | 0.1240 | 0.1157 | -0.0427 | 0.0316 | 0.3186 | -0.3162 | 0.1297 | 0.2184 | -0.1997 | 0.7341 |
| 2 | 0.1146 | 0.3114 | 0.1559 | 0.0630 | -0.1529 | -0.0351 | 0.3526 | -0.2344 | 0.0104 | 0.2737 | 0.6752 | -0.3561 |
| 3 | 0.1504 | 0.3020 | 0.1748 | 0.1031 | 0.0436 | 0.0961 | -0.0823 | -0.3943 | 0.4227 | -0.2173 | -0.4649 | -0.4791 |
| 4 | 0.1462 | 0.1164 | 0.3022 | 0.5010 | 0.2747 | -0.6416 | -0.1311 | 0.2315 | -0.1681 | 0.1525 | -0.1036 | -0.0513 |
| 5 | 0.4786 | 0.1039 | -0.5137 | 0.2285 | -0.6062 | -0.1696 | -0.0586 | 0.0685 | -0.0823 | -0.1737 | -0.0525 | -0.0008 |
| 6 | 0.2212 | 0.1967 | 0.2042 | 0.0887 | 0.2441 | 0.1806 | 0.1417 | 0.1374 | -0.1691 | -0.7809 | 0.2591 | 0.1618 |
| 7 | 0.6323 | 0.0041 | 0.1674 | -0.6874 | 0.1356 | -0.1451 | -0.0766 | 0.0071 | -0.1608 | 0.1445 | -0.0848 | -0.0203 |
| 8 | 0.1443 | 0.0350 | 0.2487 | -0.0061 | -0.1846 | 0.1488 | -0.0222 | 0.6502 | 0.6422 | 0.1063 | 0.0896 | 0.0699 |
| 9 | 0.1519 | 0.0536 | 0.0586 | 0.1686 | 0.0544 | 0.2496 | -0.8055 | -0.2510 | 0.0384 | 0.1184 | 0.3336 | 0.1956 |
| 10 | 0.0676 | 0.4076 | -0.6276 | 0.0073 | 0.5727 | 0.1249 | 0.0388 | 0.2030 | 0.1097 | 0.1823 | 0.0257 | -0.0567 |
| 11 | 0.1383 | 0.0901 | 0.1936 | 0.2384 | -0.0919 | 0.6121 | 0.0705 | 0.2314 | -0.4995 | 0.2875 | -0.2757 | -0.1696 |
| 12 | 0.4251 | -0.6808 | -0.0910 | 0.3211 | 0.2802 | 0.1406 | 0.2550 | -0.1785 | 0.1991 | 0.0704 | 0.0651 | -0.0224 |

Table S23. Variable loadings for the MAZ-1, hadrosaurid genus DFA.

|  | DF | |
| --- | --- | --- |
| PC | 1 | 2 |
| 1 | 0.99279 | -0.63136 |
| 2 | 0.49389 | -0.01857 |
| 3 | 0.95684 | 0.45839 |
| 4 | 0.021342 | 0.57246 |
| 5 | 1.0615 | 0.077951 |
| 6 | -0.62225 | -0.16457 |

Table S24. Results for the MAZ-2, suborder/family level PCA.

| PC | Eigenvalue | % variance | Cumulative % variance |
| --- | --- | --- | --- |
| 1 | 0.385902 | 79.594 | 79.594 |
| 2 | 0.073412 | 15.142 | 94.736 |
| 3 | 0.008032 | 1.6566 | 96.3926 |
| 4 | 0.006398 | 1.3195 | 97.7121 |
| 5 | 0.004546 | 0.93756 | 98.64966 |
| 6 | 0.002858 | 0.58942 | 99.23908 |
| 7 | 0.001668 | 0.34403 | 99.58311 |
| 8 | 0.000753 | 0.15534 | 99.73845 |
| 9 | 0.000504 | 0.1039 | 99.84235 |
| 10 | 0.000484 | 0.099849 | 99.9422 |
| 11 | 0.000229 | 0.047282 | 99.98948 |
| 12 | 5.51x10^-5^ | 0.011359 | 100 |

Table S25. Variable loadings for the MAZ-2, family level PCA.

|  | PC | | | | | | | | | | | |
| --- | --- | --- | --- | --- | --- | --- | --- | --- | --- | --- | --- | --- |
| Variable | 1 | 2 | 3 | 4 | 5 | 6 | 7 | 8 | 9 | 10 | 11 | 12 |
| 1 | 0.0307 | 0.2646 | 0.1691 | -0.0389 | -0.0322 | -0.0071 | 0.0969 | 0.0916 | 0.1186 | 0.1398 | 0.6434 | -0.6576 |
| 2 | -0.0066 | 0.2292 | 0.2497 | -0.0003 | 0.1017 | -0.1771 | 0.2223 | 0.0240 | 0.2842 | -0.3343 | 0.4626 | 0.6220 |
| 3 | 0.0402 | 0.2318 | 0.3126 | -0.1746 | 0.1015 | 0.0304 | -0.0108 | -0.5428 | -0.3997 | 0.5432 | 0.0779 | 0.2229 |
| 4 | 0.1542 | 0.3710 | 0.1656 | -0.4706 | -0.3894 | 0.3632 | -0.3767 | -0.0959 | 0.1363 | -0.3349 | -0.1540 | -0.0241 |
| 5 | 0.1615 | 0.2381 | -0.5521 | 0.0948 | 0.5656 | 0.4160 | -0.2052 | -0.1617 | 0.0642 | -0.0578 | 0.1805 | 0.0467 |
| 6 | -0.0475 | 0.2373 | 0.2452 | 0.0597 | 0.3067 | 0.0957 | 0.5153 | -0.2616 | 0.3768 | -0.0870 | -0.4807 | -0.2447 |
| 7 | -0.3288 | 0.5869 | -0.0146 | 0.3168 | 0.0752 | -0.4595 | -0.3515 | 0.0470 | -0.2036 | -0.1519 | -0.1783 | -0.0891 |
| 8 | 0.1509 | 0.1286 | 0.2019 | 0.1280 | 0.0860 | 0.0589 | -0.3097 | 0.4359 | 0.4772 | 0.5713 | -0.1521 | 0.1721 |
| 9 | 0.0512 | 0.1031 | 0.2700 | -0.2247 | 0.3771 | 0.2297 | 0.1646 | 0.5964 | -0.5117 | -0.1264 | -0.0882 | 0.0075 |
| 10 | 0.8791 | 0.1691 | -0.1013 | 0.1496 | -0.1137 | -0.3154 | 0.1470 | 0.0095 | -0.1343 | -0.0522 | -0.1010 | -0.0280 |
| 11 | -0.0611 | 0.1967 | 0.0387 | 0.6310 | -0.4252 | 0.5277 | 0.2207 | 0.0548 | -0.1765 | 0.0269 | 0.0392 | 0.1164 |
| 12 | -0.1875 | 0.3661 | -0.5416 | -0.3823 | -0.2525 | -0.0905 | 0.4181 | 0.2041 | 0.0251 | 0.2859 | -0.0639 | 0.1282 |

Table S26. Results for the MAZ-2, hadrosaurid genus PCA.

| PC | Eigenvalue | % variance | Cumulative % variance |
| --- | --- | --- | --- |
| 1 | 0.085941 | 68.575 | 68.575 |
| 2 | 0.023324 | 18.611 | 87.186 |
| 3 | 0.008108 | 6.4692 | 93.6552 |
| 4 | 0.003348 | 2.6714 | 96.3266 |
| 5 | 0.002108 | 1.6823 | 98.0089 |
| 6 | 0.001351 | 1.0777 | 99.0866 |
| 7 | 0.000618 | 0.49326 | 99.57986 |
| 8 | 0.000339 | 0.27031 | 99.85017 |
| 9 | 0.000176 | 0.14008 | 99.99025 |
| 10 | 1.32x10^-5^ | 0.010521 | 100 |

Table S27. Variable loadings for the MAZ-2, hadrosaurid genus PCA.

|  | PC | | | | | | | | | | | |
| --- | --- | --- | --- | --- | --- | --- | --- | --- | --- | --- | --- | --- |
| Variable | 1 | 2 | 3 | 4 | 5 | 6 | 7 | 8 | 9 | 10 | 11 | 12 |
| 1 | 0.2670 | 0.2153 | -0.0833 | 0.1738 | -0.0742 | 0.0562 | -0.4134 | 0.4825 | 0.1028 | -0.2192 | -0.5203 | -0.3216 |
| 2 | 0.2368 | 0.2077 | -0.1340 | 0.2587 | -0.2053 | 0.1221 | -0.4259 | -0.3498 | 0.5382 | 0.0206 | 0.3003 | 0.2736 |
| 3 | 0.2290 | 0.2973 | -0.1119 | -0.1036 | 0.1588 | 0.1578 | -0.1486 | 0.2660 | -0.4482 | -0.3077 | 0.6303 | -0.0101 |
| 4 | 0.3813 | 0.3008 | -0.1998 | -0.5428 | -0.1830 | -0.4616 | 0.0355 | -0.3456 | -0.0696 | 0.0573 | -0.1141 | -0.2031 |
| 5 | 0.2961 | -0.1063 | 0.9133 | -0.0787 | -0.1403 | -0.0520 | -0.1568 | -0.0321 | -0.0264 | -0.0202 | 0.0870 | -0.0631 |
| 6 | 0.2438 | 0.2401 | 0.0383 | 0.2933 | -0.2280 | 0.4898 | 0.5324 | -0.3238 | -0.1298 | -0.1936 | -0.1399 | -0.2034 |
| 7 | 0.2923 | -0.0133 | 0.0517 | -0.0597 | 0.4074 | -0.1958 | 0.3308 | 0.0890 | 0.3079 | -0.5195 | -0.1413 | 0.4493 |
| 8 | 0.2022 | 0.1078 | -0.0025 | 0.0351 | -0.0084 | 0.1361 | -0.1720 | -0.0477 | -0.4921 | 0.3049 | -0.3620 | 0.6554 |
| 9 | 0.1550 | 0.2260 | 0.0272 | -0.2114 | -0.2458 | 0.1408 | 0.3769 | 0.5509 | 0.3170 | 0.4628 | 0.1438 | 0.1353 |
| 10 | 0.5460 | -0.7564 | -0.2852 | 0.0307 | -0.1194 | 0.0747 | 0.0208 | 0.0627 | -0.0567 | 0.0710 | 0.0959 | -0.0765 |
| 11 | 0.1574 | 0.1374 | 0.0125 | 0.6741 | 0.0467 | -0.6043 | 0.1798 | 0.0850 | -0.1459 | 0.2229 | 0.1411 | -0.0631 |
| 12 | 0.2333 | 0.0939 | 0.0465 | -0.0212 | 0.7610 | 0.2323 | -0.0500 | -0.1545 | 0.1205 | 0.4289 | -0.0301 | -0.2746 |
