# Supplementary material for: Skull Ecomorphology of Megaherbivorous Dinosaurs from the Dinosaur Park Formation (Upper Campanian) of Alberta, Canada
Source: PLoS One. 2013 Jul 10;8(7):e67182. doi: 10.1371/journal.pone.0067182 (PMC3707905; doi:10.1371/journal.pone.0067182)
Supplement: Table S1 — Raw data used in this study. (DOCX) [file pone.0067182.s002.docx]

Table S1. Raw data used in this study. Taxonomic abbreviations: A, Ankylosauria; An, Ankylosauridae; C, Ceratopsidae; Ce, Centrosaurinae; Cen, *Centrosaurus*; Ch, Chasmosaurinae; Cha, *Chasmosaurus*; Cor, *Corythosaurus*; Euo, *Euoplocephalus*; Gry, *Gryposaurus*; H, Hadrosauridae; Ha, Hadrosaurinae; La, Lambeosaurinae; Lam, *Lambeosaurus*; No, Nodosauridae; pac, ‘pachyrhinosaur’; Pan, *Panoplosaurus*; Par, *Parasaurolophus*; Pro, *Prosaurolophus*; Sty, *Styracosaurus*; Vag, *Vagaceratops*. Other abbreviations: MAZ, Megaherbivore Assemblage Zone. Estimated values listed in bold. See Figure 1 and Table 1 for variable descriptions.

|  |  |  |  | Variable | | | | | | | | | | | |  |
| --- | --- | --- | --- | --- | --- | --- | --- | --- | --- | --- | --- | --- | --- | --- | --- | --- |
| Suborder/  family | Family/  subfamily | Genus | Specimen | 1 | 2 | 3 | 4 | 5 | 6 | 7 | 8 | 9 | 10 | 11 | 12 | MAZ |
| A | An | Euo | [AMNH 5337](http://paleo.amnh.org/search.php?action=detail&specimen_id=48103) | 290 | 210 | 207 | 85 | 185 | 80 | 268 | 63 | **50** | 33 | 133 | 251 | ? |
| A | An | Euo | [AMNH 5403](http://paleo.amnh.org/search.php?action=detail&specimen_id=48176) | ? | **213** | **229** | **104** | 235 | 69 | 286 | 50 | 61 | 34 | 104 | 261 | ? |
| A | An | Euo | [AMNH 5405](http://paleo.amnh.org/search.php?action=detail&specimen_id=48178) | 278 | 173 | 184 | 77 | 180 | 67 | 250 | **67** | 45 | 35 | 116 | 225 | ? |
| A | An | Euo | CMN 8876 | **266** | 199 | 202 | 84 | 171 | ? | 285 | **62** | ? | 36 | **103** | 223 | ? |
| A | An | Euo | NHMUK R4947 | 280 | 190 | 185 | 61 | 170 | ? | **236** | 59 | ? | 30 | 127 | **230** | ? |
| A | An | Euo | ROM 1930 | 280 | 186 | 216 | 90 | 210 | ? | 241 | 62 | ? | 48 | 171 | 240 | ? |
| A | An | Euo | [TMP 1991.127.0001](http://hermis.cd.gov.ab.ca/rtmp/Details.aspx?ObjectID=TMP1991.127.0001&dv=True) | 232 | 156 | 148 | 72 | 160 | ? | 211 | 57 | ? | 31 | 78 | 182 | 1 |
| A | An | Euo | [TMP 1997.132.0001](http://hermis.cd.gov.ab.ca/rtmp/Details.aspx?ObjectID=TMP1997.132.0001&dv=True) | 293 | 186 | 208 | 78 | 170 | 76 | 258 | 55 | 69 | 36 | 111 | 260 | ? |
| A | An | Euo | [UALVP 31](http://www.museums.ualberta.ca/dig/search/vpaleo/search_details.php?&index=0&totalcount=1&ff0=UALVP31&result=/dig/search/vpaleo/search_results.php%3Fclass%3D%26sciname%3D%26family%3D%26status%3D%26d1%3D%26d2%3D%26formation%3D%26locality%3D%26age%3D%26sp1%25) | 285 | 209 | 178 | 115 | 182 | 89 | 244 | 42 | 57 | 40 | 91 | 249 | 2 |
| A | No | Pan | [AMNH 5381](http://paleo.amnh.org/search.php?action=detail&specimen_id=48151) | 331 | 229 | ? | **115** | 132 | ? | 271 | 65 | 115 | 24 | **105** | 230 | 1 |
| A | No | Pan | CMN 2759 | 295 | 213 | 184 | 84 | 127 | 56 | 218 | 47 | 91 | 45 | 94 | 190 | 1 |
| A | No | Pan | [ROM 1215](http://piclib.nhm.ac.uk/piclib/webimages/0/3000/900/3988_big.jpg) | 300 | 208 | 174 | 81 | 131 | 58 | 214 | 46 | 65 | 29 | 90 | 235 | 1 |
| A | No | Pan | [TMP 1983.025.0002](http://hermis.cd.gov.ab.ca/rtmp/Details.aspx?ObjectID=TMP1983.025.0002&dv=True) | **334** | **210** | **220** | **104** | 159 | ? | **178** | 37 | ? | 29 | ? | **140** | ? |
| A | No | Pan | [TMP 1998.074.0001](http://hermis.cd.gov.ab.ca/rtmp/Details.aspx?ObjectID=TMP1998.074.0001&dv=True) | 342 | 255 | 259 | **115** | 154 | ? | 256 | 70 | ? | 21 | 112 | **254** | 1 |
| A | No | Pan | [TMP 1998.098.0001](http://hermis.cd.gov.ab.ca/rtmp/Details.aspx?ObjectID=TMP1998.098.0001&dv=True) | 380 | 252 | 260 | 124 | 162 | 77 | ? | ? | 92 | 43 | 112 | **244** | 1 |
| A | No | Pan | [TMP 2000.012.0158](http://hermis.cd.gov.ab.ca/rtmp/Details.aspx?ObjectID=TMP2000.012.0158&dv=True) | 365 | 255 | 260 | 143 | 139 | ? | 233 | 91 | ? | 20 | 134 | **235** | ? |
| A | No | Pan | [UALVP 16249](http://www.museums.ualberta.ca/dig/search/vpaleo/search_details.php?&index=0&totalcount=1&ff0=UALVP16249&result=/dig/search/vpaleo/search_results.php%3Fclass%3D%26sciname%3D%26family%3D%26status%3D%26d1%3D%26d2%3D%26formation%3D%26locality%3D%26age%3D%26s) | 405 | 299 | 285 | 120 | **190** | ? | 261 | 70 | ? | 30 | 127 | 237 | ? |
| C | Ce | Cen | [AMNH 5351](http://paleo.amnh.org/search.php?action=detail&specimen_id=48119) | 637 | 507 | 330 | 42 | 128 | 126 | 371 | 96 | 180 | 14 | 385 | 199 | 1 |
| C | Ce | Cen | [AMNH 5377](http://paleo.amnh.org/search.php?action=detail&specimen_id=48147) | ? | ? | **475** | **50** | ? | 102 | 390 | 193 | 187 | ? | 511 | 310 | ? |

**Table S1. (Continued)**

|  |  |  |  | Variable | | | | | | | | | | | |  |
| --- | --- | --- | --- | --- | --- | --- | --- | --- | --- | --- | --- | --- | --- | --- | --- | --- |
| Suborder/  family | Family/  subfamily | Genus | Specimen | 1 | 2 | 3 | 4 | 5 | 6 | 7 | 8 | 9 | 10 | 11 | 12 | MAZ |
| C | Ce | Cen | CMN 348 | 723 | 573 | 409 | 85 | 89 | 124 | **448** | 151 | 225 | 63 | 361 | 295 | ? |
| C | Ce | Cen | CMN 8790 | ? | **580** | 313 | **60** | ? | ? | **431** | 63 | ? | ? | 247 | ? | ? |
| C | Ce | Cen | CMN 8795 | 664 | 504 | 356 | 60 | 120 | 115 | 489 | 175 | 168 | 40 | 310 | 322 | 1 |
| C | Ce | Cen | [NHMUK R4859](http://piclib.nhm.ac.uk/piclib/webimages/0/4000/700/4727_big.jpg) | **656** | **495** | **345** | **30** | **110** | ? | **480** | 127 | ? | 20 | 340 | **200** | ? |
| C | Ce | Cen | [ROM 767](http://images.rom.on.ca/public/images/ROM2007_9464_8.jpg) | 652 | 498 | 353 | 56 | 97 | 105 | 352 | 127 | 186 | 37 | 352 | 190 | 1 |
| C | Ce | Cen | [TMP 1997.085.0001](http://hermis.cd.gov.ab.ca/rtmp/Details.aspx?ObjectID=TMP1997.085.0001&dv=True) | **735** | **544** | **371** | **74** | 173 | 136 | ? | ? | 164 | 72 | 273 | **525** | 1 |
| C | Ce | Cen | [UALVP 11735](http://www.museums.ualberta.ca/dig/search/vpaleo/search_details.php?&index=1&totalcount=6&ff0=UALVP41&ff1=UALVP11735&ff2=UALVP16248&ff3=UALVP47967&ff4=UALVP47979&ff5=UALVP49336&result=/dig/search/vpaleo/search_results.php%3Fclass%3D%26sciname%3Dcentrosaur) | 592 | 440 | 293 | 35 | 147 | ? | 499 | 90 | ? | 32 | 261 | 270 | 1 |
| C | Ce | Cen | [UALVP 16248](http://www.museums.ualberta.ca/dig/search/vpaleo/search_details.php?&index=2&totalcount=6&ff0=UALVP41&ff1=UALVP11735&ff2=UALVP16248&ff3=UALVP47967&ff4=UALVP47979&ff5=UALVP49336&result=/dig/search/vpaleo/search_results.php%3Fclass%3D%26sciname%3Dcentrosaur) | 540 | 424 | 320 | ? | 95 | 95 | **360** | 140 | 140 | 29 | 340 | 214 | 1 |
| C | Ce | Cen | [USNM 8897](http://nhb-acsmith1.si.edu/emuwebpalweb/pages/nmnh/pal/Display.php?irn=3383668&QueryPage=/emuwebpalweb/pages/nmnh/pal/Query.php) | 665 | 491 | 336 | 61 | 101 | 108 | 392 | 147 | 181 | 31 | 384 | 230 | 1 |
| C | Ce | Cen | [YPM 2015](http://research.yale.edu/cgi-bin/cgiwrap/ypm3/Query.Ledger?LE=vp&ST=2&TX=&TA=&HT=&TS=&CI=&LO=&CO=&PE=&OT=&ID=2015&LI=&SO=1) | 676 | 523 | 365 | 74 | 129 | 113 | 451 | 158 | 199 | 73 | 400 | 325 | 1 |
| C | Ce | pac | [TMP 2002.076.0001](http://hermis.cd.gov.ab.ca/rtmp/Details.aspx?ObjectID=TMP2002.076.0001&dv=True) | 750 | 548 | 406 | 100 | 125 | 119 | ? | ? | 186 | 20 | 519 | 320 | 2 |
| C | Ce | Sty | [CMN 344](http://www.expressnews.ualberta.ca/images/8014.001.jpg) | ? | ? | 307 | 53 | ? | 116 | 564 | 98 | 203 | ? | 319 | 409 | 2 |
| C | Ch | Cha | CMN 2245 | ? | ? | ? | **105** | ? | 128 | **508** | 150 | 195 | ? | 305 | 317 | 2 |
| C | Ch | Cha | ROM 839 | ? | 565 | 398 | 49 | ? | 133 | **430** | 120 | 218 | ? | 346 | 186 | 2 |
| C | Ch | Cha | [ROM 843](http://images.rom.on.ca/public/images/ROM2007_9608_9.jpg) | ? | ? | 424 | 68 | ? | 140 | 645 | 80 | 200 | ? | 313 | 530 | 2 |
| C | Ch | Cha | [YPM 2016](http://research.yale.edu/cgi-bin/cgiwrap/ypm3/Query.Ledger?LE=vp&ST=2&TX=&TA=&HT=&TS=&CI=&LO=&CO=&PE=&OT=&ID=2016&LI=&SO=1) | 703 | 558 | 386 | 43 | 101 | ? | 558 | 110 | ? | 25 | 246 | 375 | 1 |
| C | Ch | Cha | CMN 2280 | 685 | 512 | 364 | 70 | 102 | 116 | **470** | 140 | 170 | 60 | 300 | 306 | 1 |
| C | Ch | Cha | CMN 8801 | 840 | 666 | **465** | **109** | 103 | 137 | ? | ? | ? | 14 | 453 | ? | 1 |
| C | Ch | Cha | [TMP 1981.019.0175](http://hermis.cd.gov.ab.ca/rtmp/Details.aspx?ObjectID=TMP1981.019.0175&dv=True) | 695 | 570 | 358 | 69 | 101 | ? | **502** | 131 | ? | 18 | 313 | **360** | 1 |
| C | Ch | Cha | [UALVP 40](http://www.museums.ualberta.ca/dig/search/vpaleo/search_details.php?&index=0&totalcount=1&ff0=UALVP40&result=/dig/search/vpaleo/search_results.php%3Fclass%3D%26sciname%3DChasmosaurus%26family%3D%26status%3D%26d1%3D%26d2%3D%26formation%3D%26locality%3D%26a) | ? | 458 | 315 | 39 | ? | 99 | **350** | 104 | ? | ? | 261 | **180** | ? |
| C | Ch | Vag | CMN 41357 | 703 | 568 | ? | 79 | 124 | 107 | ? | ? | 198 | 72 | 319 | 340 | ? |
| H | Ha | Gry | CMN 2278 | 796 | 615 | 521 | **140** | 238 | 142 | 423 | 211 | **289** | 89 | 337 | **318** | 1 |
| H | Ha | Gry | [ROM 764](http://images.rom.on.ca/public/images/ROM2007_9608_46.jpg) | ? | ? | ? | **125** | ? | 105 | **376** | 148 | **245** | ? | 223 | **236** | 1 |
| H | Ha | Gry | ROM 873 | 791 | 583 | 530 | 134 | 201 | 125 | **176** | **146** | 284 | 107 | 305 | 190 | 1 |

**Table S1. (Continued)**

|  |  |  |  | Variable | | | | | | | | | | | |  |
| --- | --- | --- | --- | --- | --- | --- | --- | --- | --- | --- | --- | --- | --- | --- | --- | --- |
| Suborder/  family | Family/  subfamily | Genus | Specimen | 1 | 2 | 3 | 4 | 5 | 6 | 7 | 8 | 9 | 10 | 11 | 12 | MAZ |
| H | Ha | Gry | [TMP 1980.022.0001](https://hermis.alberta.ca/rtmp/Details.aspx?ObjectID=TMP1980.022.0001&dv=True) | 580 | 440 | 382 | 105 | **94** | 92 | 197 | 153 | **220** | 75 | 238 | **174** | 1 |
| H | Ha | Gry | TMP 1991.081.0001 | **560** | **410** | **355** | **55** | ? | 91 | **150** | ? | **217** | 97 | 243 | ? | ? |
| H | Ha | Pro | CMN 2277 | **799** | **670** | **498** | **116** | 130 | 131 | ? | ? | 254 | 62 | 296 | 235 | 2 |
| H | Ha | Pro | CMN 2870 | 803 | 623 | 512 | 98 | 221 | 129 | **270** | 142 | **258** | 67 | 295 | 330 | ? |
| H | Ha | Pro | ROM 1928 | **663** | **501** | **407** | **93** | ? | 96 | **152** | ? | **247** | 76 | 218 | ? | 2 |
| H | Ha | Pro | [ROM 787](http://images.rom.on.ca/public/images/ROM2008_9677_1.jpg) | 793 | 609 | 488 | **124** | 166 | 133 | 170 | 183 | 271 | 79 | 302 | 200 | 2 |
| H | Ha | Pro | [TMM 41262](http://www.npl.utexas.edu/vpl/databases/index.php?page=1&page_size=10&mode=search&action=search_record&searchtype=&specimen_id=36696&taxon=prosaurolophus&locality=&county=&state=&specid=&geol_form=&epoch=&site_no=&showimage=&geog=&country=&others=&geol=&e) | 639 | 499 | 419 | 114 | **104** | 91 | ? | ? | **223** | 110 | 222 | ? | 2 |
| H | Ha | Pro | [TMP 1984.001.0001](https://hermis.alberta.ca/rtmp/Details.aspx?ObjectID=TMP1984.001.0001&dv=True) | 900 | 650 | 535 | 140 | **248** | 139 | ? | ? | 294 | 125 | 280 | ? | 2 |
| H | Ha | Pro | [USNM 12712](http://nhb-acsmith1.si.edu/emuwebpalweb/pages/nmnh/pal/Display.php?irn=3440890&QueryPage=/emuwebpalweb/pages/nmnh/pal/Query.php) | 975 | 699 | 575 | 140 | **130** | 123 | ? | **190** | 272 | 83 | 348 | **240** | 2 |
| H | La | Cor | FMNH 1357 | 589 | 462 | **389** | **102** | ? | 97 | ? | ? | **235** | 120 | 255 | ? | ? |
| H | La | Cor | ROM 1933 | 617 | 480 | 365 | 80 | 147 | 87 | **160** | 123 | 224 | 107 | 230 | ? | 1 |
| H | La | Cor | ROM 870 | ? | ? | 339 | 97 | ? | 75 | **94** | ? | 195 | ? | 212 | **134** | 1 |
| H | La | Cor | ROM 871 | ? | ? | ? | 100 | ? | 108 | **188** | 156 | 225 | ? | 275 | 75 | 1 |
| H | La | Cor | [TMP 1980.040.0001](https://hermis.alberta.ca/rtmp/Details.aspx?ObjectID=TMP1980.040.0001&dv=True) | 575 | 457 | 355 | 114 | 209 | 90 | 262 | 169 | **230** | 53 | 266 | 363 | 1 |
| H | La | Cor | [TMP 1984.121.0001](https://hermis.alberta.ca/rtmp/Details.aspx?ObjectID=TMP1984.121.0001&dv=True) | 630 | 507 | 384 | **99** | 215 | 92 | **222** | **200** | **235** | 44 | 260 | 285 | ? |
| H | La | Cor | CMN 8676 | 564 | 434 | 338 | 90 | 141 | 81 | 151 | 140 | 179 | 74 | 213 | **300** | 1 |
| H | La | Cor | CMN 8704 | 596 | 430 | 385 | 82 | 120 | 84 | ? | **145** | 233 | 77 | 271 | 220 | 1 |
| H | La | Cor | ROM 776 | 657 | 489 | 407 | 109 | **220** | 83 | 170 | 160 | 271 | 91 | 270 | **256** | 1 |
| H | La | Cor | ROM 777 | 683 | 542 | 405 | 100 | 173 | 82 | ? | ? | 237 | 76 | 260 | **190** | 1 |
| H | La | Cor | [ROM 845](http://images.rom.on.ca/public/images/ROM2007_9608_32.jpg) | 630 | 491 | 369 | **100** | 220 | 90 | 205 | 135 | **230** | 100 | 273 | 247 | 1 |
| H | La | Cor | [TMP 1980.023.0004](https://hermis.alberta.ca/rtmp/Details.aspx?ObjectID=TMP1980.023.0004&dv=True) | ? | ? | 426 | **86** | ? | ? | **212** | ? | **260** | ? | 262 | 278 | 1 |
| H | La | Cor | ROM 868 | **691** | **535** | 406 | 112 | **124** | 96 | ? | ? | 226 | ? | 292 | **190** | 1 |
| H | La | Cor | [TMP 1982.037.0001](https://hermis.alberta.ca/rtmp/Details.aspx?ObjectID=TMP1982.037.0001&dv=True) | 529 | 419 | 344 | 93 | 136 | 74 | **122** | 141 | 214 | 65 | 221 | **150** | 1 |
| H | La | Cor | [TMP 1997.012.0232](https://hermis.alberta.ca/rtmp/Details.aspx?ObjectID=TMP1997.012.0232&dv=True) | **460** | **320** | **287** | **56** | ? | 68 | ? | ? | **170** | 55 | 192 | ? | ? |

**Table S1. (Continued)**

|  |  |  |  | Variable | | | | | | | | | | | |  |
| --- | --- | --- | --- | --- | --- | --- | --- | --- | --- | --- | --- | --- | --- | --- | --- | --- |
| Suborder/  family | Family/  subfamily | Genus | Specimen | 1 | 2 | 3 | 4 | 5 | 6 | 7 | 8 | 9 | 10 | 11 | 12 | MAZ |
| H | La | Lam | CMN 8703 | 671 | 552 | 399 | 90 | 160 | 94 | **186** | **160** | 216 | 59 | 310 | **190** | 1 |
| H | La | Lam | [ROM 869](http://images.rom.on.ca/public/images/ROM2004_1028_16.jpg) | 530 | 444 | 340 | **104** | 114 | 75 | **120** | ? | **212** | 80 | 218 | **180** | 1 |
| H | La | Lam | [TMP 1981.037.0001](https://hermis.alberta.ca/rtmp/Details.aspx?ObjectID=TMP1981.037.0001&dv=True) | 664 | 540 | 409 | 82 | 202 | 92 | 205 | 175 | 227 | 93 | 258 | 155 | 1 |
| H | La | Lam | CMN 2869 | 598 | 456 | 348 | 106 | 130 | 89 | **142** | ? | 191 | 52 | 256 | **248** | ? |
| H | La | Lam | FMNH 1479 | 681 | 507 | 400 | 107 | **226** | 95 | **160** | 148 | 212 | 128 | 263 | **190** | 1 |
| H | La | Lam | [ROM 1218](http://images.rom.on.ca/public/images/ROM2007_9608_47.jpg) | 682 | 498 | 422 | **95** | **196** | 101 | 205 | 168 | **216** | 114 | 282 | 257 | 2 |
| H | La | Lam | ROM 794 | 705 | 569 | 364 | 95 | 182 | 99 | **174** | 162 | 210 | 153 | 290 | 185 | 2 |
| H | La | Lam | [TMP 1982.038.0001](https://hermis.alberta.ca/rtmp/Details.aspx?ObjectID=TMP1982.038.0001&dv=True) | 492 | 378 | 321 | **77** | 215 | 85 | 280 | 140 | **238** | 98 | 247 | 375 | 1 |
| H | La | Lam | [TMP 1997.012.0128](https://hermis.alberta.ca/rtmp/Details.aspx?ObjectID=TMP1997.012.0128&dv=True) | 605 | 472 | 350 | 67 | **110** | 100 | ? | ? | 207 | 90 | 271 | ? | 2 |
| H | La | Lam | CMN 8705 | 668 | 507 | 413 | **113** | **152** | 102 | **176** | 152 | **256** | 65 | 290 | **192** | 2 |
| H | La | Lam | [TMP 1966.004.0001](https://hermis.alberta.ca/rtmp/Details.aspx?ObjectID=TMP1966.004.0001&dv=True) | 634 | 496 | 378 | 93 | 140 | 100 | **142** | ? | **225** | 91 | 265 | 116 | ? |
| H | La | Lam | CMN 351 | 679 | **552** | 400 | 105 | **116** | 89 | ? | ? | 235 | 32 | 279 | ? | ? |
| H | La | Lam | CMN 8503 | 469 | 372 | 305 | **55** | 104 | 72 | **102** | 119 | **186** | 28 | 211 | 140 | 2 |
| H | La | Lam | NHMUK R9527 | 591 | ? | 404 | 100 | ? | 87 | ? | ? | **245** | 36 | 271 | ? | ? |
| H | La | Par | [ROM 768](http://images.rom.on.ca/public/images/ROM2007_9608_45.jpg) | 637 | 486 | 404 | **113** | **180** | 102 | **192** | ? | **235** | 125 | 229 | **185** | 1 |
